# Vacancy-enhanced generation of singlet oxygen for photodynamic therapy†
†Electronic supplementary information (ESI) available. See DOI: 10.1039/c8sc05275a


**DOI:** 10.1039/c8sc05275a

**Published:** 2018-12-20

**Authors:** Shanyue Guan, Li Wang, Si-Min Xu, Di Yang, Geoffrey I. N. Waterhouse, Xiaozhong Qu, Shuyun Zhou

**Affiliations:** a Key Laboratory of Photochemical Conversion and Optoelectronic Materials , Technical Institute of Physics and Chemistry , Chinese Academy of Sciences , Beijing , 100190 , P. R. China . Email: zhou_shuyun@mail.ipc.ac.cn; b College of Materials Science and Opto-Electronic Technology, University of Chinese Academy of Sciences , Beijing 100049 , China . Email: quxz@iccas.ac.cn; c State Key Laboratory of Chemical Resource Engineering , Beijing University of Chemical Technology , 100029 , Beijing , P. R. China; d School of Chemical Sciences , The University of Auckland , Auckland 1142 , New Zealand

## Abstract

Oxygen vacancy (OV) engineering in semiconductors can greatly enhance the separation of photo-induced electron–hole pairs, thereby enhancing the photocatalytic activity. Taking inspiration from this, we prepared a novel BiOBr–H/Rub_2_d composite by functionalizing OV-rich BiOBr (named BiOBr–H) with a carboxyl functionalized ruthenium photosensitizer (Ru(bpy)_2_C-pyCl_2_, abbreviated as Rub_2_d), which was then successfully applied for photodynamic therapy (PDT). Density functional theory (DFT) calculations confirmed efficient electron transfer from the Rub_2_d complex to the intermediate energy level of BiOBr–H under visible light irradiation. *In vitro* and *in vivo* studies demonstrated that BiOBr–H/Rub_2_d was a superior agent for photodynamic therapy compared with the free ruthenium complex. The theoretical and experimental data presented thus reveal for the first time that abundant OVs in BiOBr–H can significantly improve the photocatalytic activity of a photosensitizer, resulting in the generation of more reactive oxygen species to enhance PDT. The findings of this study thus offer a new strategy for the development of highly efficient cancer therapies.

## Abstract 2

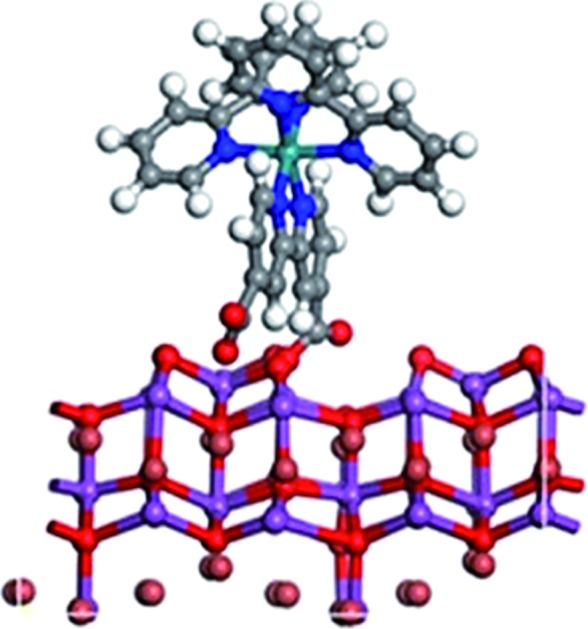
Oxygen vacancy introduced defects in the band gap of BiOBr–H allow facile electron transfer from a photo-excited ruthenium complex to the semiconductor, thereby increasing ROS yields and PDT efficiency.

## Introduction

Cancer has always been one of the most common diseases in humans, which has been a threat to human beings.^1^,^2^ Photodynamic therapy (PDT) has been widely applied in cancer treatment due to its non-invasive properties, few side effects, easy procedure and short treatment time relative to surgery or chemotherapy.^3^,^4^ In the PDT system, the mechanism is driven *via* the excitation of a photosensitizer (PS) to its triplet state, from which energy is then transferred to triplet oxygen, leading to the production of singlet oxygen (^1^O_2_).^5^–^7^ Over the past decade, researchers have developed various PSs for PDT, including indocyanine green (ICG),^8^ chlorin e6 (Ce6),^9^–^12^ zinc phthalocyanine (ZnPc),^13^ and ruthenium complexes.^14^,^15^ However, traditional PSs suffer from photo-bleaching under irradiation, resulting in the recombination of electron–hole pairs, leading to low production rates of singlet oxygen.^16^ Therefore, it is highly desirable to realize the reaction mechanism and discover a method that can prevent the recombination of electron–hole pairs and improve the PDT therapeutic effect.

As far as we are concerned, oxygen vacancies (OVs), *i.e.*, the number of oxygen atoms expected in a compound is less (or missing) than what it should be in its perfect crystal lattice,^17^ can facilitate photogenerated charge separation in semiconductors.^18^,^19^ As a result, OVs can offer more carriers for surface reactions that lead to the generation of reactive oxygen species (ROS), such as ^1^O_2_, H_2_O_2_, ·O_2_^–^, ·OH, *etc.*^20^–^22^ OVs have always been considered as an effective electron trap since they are electron-deficient.^23^,^24^ Recently, many semiconductors have been engineered with abundant OVs, including TiO_2_, MnO_2_ and BiOBr.^25^,^26^ By virtue of the abundant OVs, low toxicity, moderate band gap and outstanding photostability properties of BiOBr,^17^ we meticulously designed a novel composite by functionalizing OV-rich BiOBr (denoted as BiOBr–H) with a PS (ruthenium complexes). Under this circumstance, the generation of ROS (not only singlet oxygen but also superoxide and hydroxyl radicals) can be considerably enhanced, leading to outstanding PDT performance. This method can not only be applied in this specific system but also provide a mechanism for other PSs, resulting in a better understanding of the oxygen vacancy engineering PDT process.

Herein, a BiOBr–H/Rub_2_d composite was successfully prepared *via* the interaction between BiOBr–H and a ruthenium complex PS (di(2,2′-bipyridine) 2,2′-bipyridine-4,4′-dicarboxyl dichlororuthenium(ii), denoted herein as Rub_2_d), as detailed in the ESI.† Compared with the Rub_2_d complex alone, the BiOBr–H/Rub_2_d agent can considerably improve the production of ROS under light irradiation, which can be verified by both ESR data and the DFT calculation. Specifically, the ^1^O_2_ yield of BiOBr–H/Rub_2_d (0.49) was more than twice that of Rub_2_d (0.22). Furthermore, both *in vitro* and *in vivo* studies confirmed that the BiOBr–H/Rub_2_d composite was a potent PDT agent for cancer treatment. Density functional theory (DFT) calculations established that the photogenerated electrons of Rub_2_d can be facilely transferred to the intermediate energy level located in the forbidden zone of BiOBr–H, facilitating electron–hole separation in BiOBr–H/Rub_2_d and thus enhancing the ^1^O_2_ yield. To demonstrate the general applicability of this strategy, here we additionally designed and synthesized two BiOBr–H/PS composites that bind specifically with the photosensitizers indocyanine green (ICG) and zinc phthalocyanine (ZnPc). As illustrated in our strategy, compared with the PS complex, the BiOBr–H/PS composite significantly increased the generation of ^1^O_2_ under irradiation. Thus, this strategy of OV-enhanced generation of singlet oxygen holds great potential in the precise treatment of cancer.

## Results and discussion

### Structural and morphological characterization

**Scheme 1:**
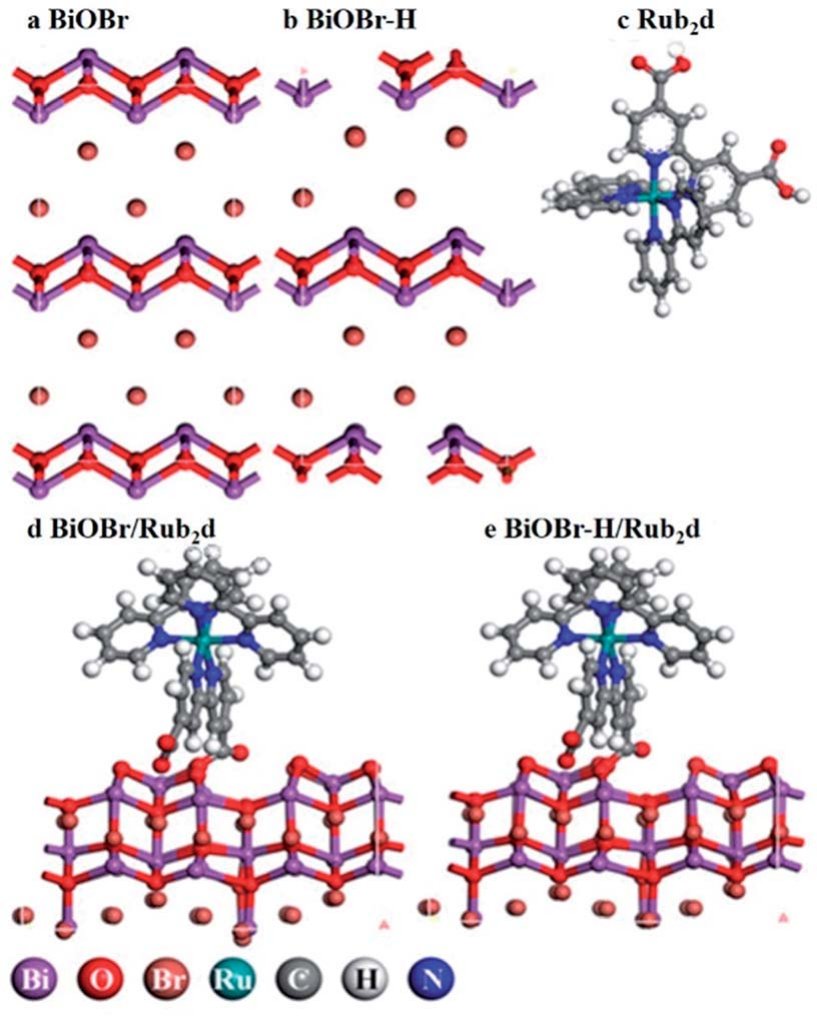
Schematic illustration of the optimized geometries of BiOBr, BiOBr–H, Rub_2_d, BiOBr/Rub_2_d, and BiOBr–H/Rub_2_d. The detailed chemical structure of Rub_2_d is listed in Fig. S1.†

BiOBr was fabricated *via* a hydrothermal method,^27^ followed by heating in O_2_ for 4 h (the product was denoted as BiOBr–H). The hydrothermally synthesized BiOBr without calcination was employed here for comparison purposes in PDT tests (and denoted simply as BiOBr). The optimized geometries of the models BiOBr, BiOBr–H, Rub_2_d, BiOBr/Rub_2_d, and BiOBr–H/Rub_2_d are displayed in Scheme 1. Ru(bpy)_2_C-pyCl_2_ (denoted as Rub_2_d) was prepared *via* a two-step reaction of ruthenium chloride with 2,2′-bipyridine and 2,2′-bipyridine-4,4′-dicarboxylic acid, the molecular structure of which is shown in Fig. S1.† BiOBr–H/Rub_2_d and BiOBr/Rub_2_d were synthesized *via* the electrostatic attraction between positively charged BiOBr–H (zeta potential +12.60 mV) and negatively charged Ru(bpy)_2_C-pyCl_2_ (zeta potential –0.97 mV). The zeta potential of the resulting composite product is measured to be +12.30 mV (Fig. S2†).

**Fig. 1:**
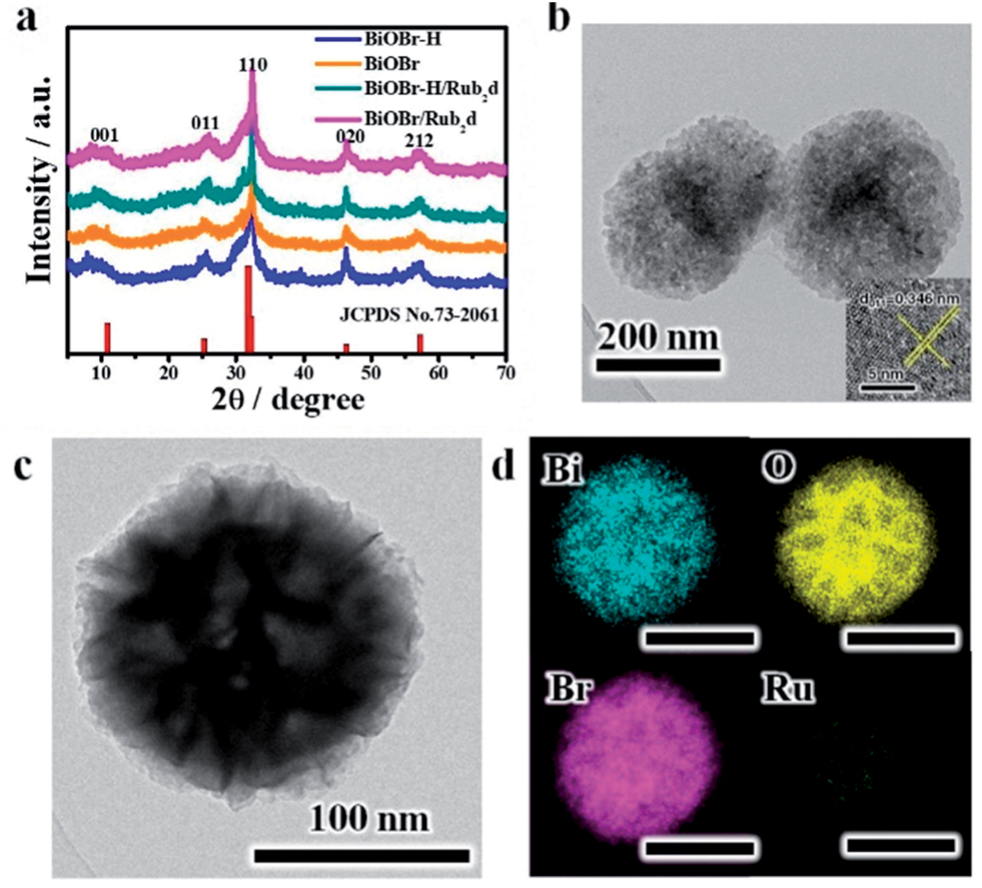
(a) XRD patterns for BiOBr, BiOBr/Rub_2_d, BiOBr–H and BiOBr–H/Rub_2_d; the standard PDF card for BiOBr is also shown (JCPDS no. 73-2061). (b) TEM image (inset: HRTEM image) and (c and d) elemental maps for BiOBr–H/Rub_2_d.

Powder X-ray diffraction (XRD) was used to characterize the structures of the various samples (Fig. 1a). BiOBr, BiOBr–H, BiOBr/Rub_2_d and BiOBr–H/Rub_2_d all showed diffraction patterns typical of BiOBr (JCPDS, PDF #73-2061). Transmission electron microscopy (TEM) and elemental mapping (EM) were used to examine the compositional uniformity of BiOBr–H/Rub_2_d. TEM (Fig. 1b and c) revealed that BiOBr–H/Rub_2_d possessed a plate-like shape with a diameter of around 200 nm. The lattice fringe spacing of 0.346 nm can be attributed to the (011) plane of BiOBr–H (Fig. 1b). Furthermore, TEM images of BiOBr–H were recorded after incubation in the cell culture medium (DMEM) or buffer solution (PBS) pH 6.5 with H_2_O_2_ (tumor microenvironment) for 24 h. As shown in Fig. S3† the morphology of BiOBr–H showed no apparent change after 24 h of incubation, indicating that BiOBr–H was stable in the acidic environment and cell culture medium. Atomic force microscopy (AFM) determined the thickness of the sheets in BiOBr–H/Rub_2_d to be approximately 1.0 nm (Fig. S4†). Elemental mapping images demonstrate that the Rub_2_d complex was uniformly distributed over the BiOBr–H surface (Fig. 1d).

UV-vis absorption spectra of BiOBr–H, BiOBr, Rub_2_d, BiOBr/Rub_2_d and BiOBr–H/Rub_2_d are presented in Fig. S5.† BiOBr and BiOBr–H displayed weak absorption across the 300–900 nm region. After functionalization with the Rub_2_d complex, a strong absorption signal at 460 nm appeared, which can readily be attributed to metal-to-ligand charge-transfer (MLCT) transitions in the Rub_2_d complex.^28^ Fourier transform infrared (FT-IR) spectroscopy was utilized to analyze the functional groups present in the various samples. For pristine BiOBr and BiOBr–H (Fig. S6†), peaks at 527 cm^–1^ and 3445 cm^–1^ were observed and assigned to the Bi–O stretching and O–H stretching modes, respectively. The peak at 2870 cm^–1^ is a C–H stretching vibration of polyvinyl pyrrolidone (PVP).^29^ This feature was much weaker for BiOBr–H. The Rub_2_d complex showed a strong peak around 1700 cm^–1^ due to the carboxylate groups of the C-py ligand^30^ (Fig. S6†). The spectra of BiOBr/Rub_2_d and BiOBr–H/Rub_2_d also show the IR peaks of the Rub_2_d complex, confirming the successful loading of the Rub_2_d complex onto these materials.

The chemical composition of BiOBr, BiOBr–H, Rub_2_d, and BiOBr–H/Rub_2_d was further probed by X-ray photoelectron spectroscopy (XPS) (Fig. S7–S10 and Table S1†). Comparing the spectra of BiOBr and BiOBr–H, the most obvious difference is the absence of the C 1s and N 1s signals in the survey spectrum of BiOBr–H. This is explained by the decomposition of PVP during the calcination step used to synthesize BiOBr–H. The spectrum of the Rub_2_d complex displayed signals due to Ru, O, N and C, whilst that of the BiOBr–H/Rub_2_d complex contained signals due to C, O, Br, Ru, N and Bi elements (Fig. S7–S8†). High-resolution Bi 4f XPS spectra of BiOBr and BiOBr–H (Fig. S9†) showed two peaks at 158.9 and 164.2 eV in a 4 : 3 area ratio, which can readily be assigned to the Bi 4f_7/2_ and 4f_5/2_ peaks of Bi^3+^.^26^ For BiOBr–H, two additional peaks are seen at 157.8 and 163.5 eV which are assigned to the Bi 4f_7/2_ and 4f_5/2_ peaks of Bi^3+^ near oxygen vacancies.^26^ The O 1s spectrum (Fig. S10†) of BiOBr–H was deconvoluted into two peaks at 529.7 and 530.9 eV. The intense peak at 529.7 eV is typical of lattice oxygen (O^2–^) in BiOBr–H, whereas the weaker peak at higher binding energy is due to the adsorbed hydroxyl species or oxygen species at vacancy sites (O^–^).^19^ The combination of the Bi 4f and O 1s data provides good evidence for the presence of OVs in the BiOBr–H sample.

Due to the intrinsic fluorescence properties of the Rub_2_d complex, we probed the fluorescence properties of Rub_2_d, BiOBr–H/Rub_2_d and BiOBr/Rub_2_d. All samples displayed an obvious fluorescence signal centered at 650 nm (similar to Rub_2_d at a concentration of 250 μg mL^–1^) (Fig. S11†). To further characterize the fluorescence performance of the samples, the two-photon fluorescence intensities of BiOBr, BiOBr–H, Rub_2_d, BiOBr/Rub_2_d and BiOBr–H/Rub_2_d were examined under 800 nm irradiation (Fig. S12†). Rub_2_d exhibited a higher up-conversion photoluminescence (UCPL) intensity than BiOBr–H/Rub_2_d, whilst no two-photon signal was seen for BiOBr and BiOBr–H. For BiOBr–H/Rub_2_d, a quadratic relationship was found between the laser power and the fluorescence intensity, confirming that the up-conversion photoluminescence emission of the sample was due to a two-photon excitation process (Fig. S13†). The emission lifetime of Rub_2_d and BiOBr–H/Rub_2_d was further examined under 520 nm excitation. The emission lifetime of BiOBr–H/Rub_2_d was 309.44 ns, longer than that of pristine Rub_2_d in solution (234.47 ns) (Fig. S14 and Table S2†). This proves conclusively that the triple state of Rub_2_d was enhanced by interaction with BiOBr–H.^31^,^32^

**Fig. 2:**
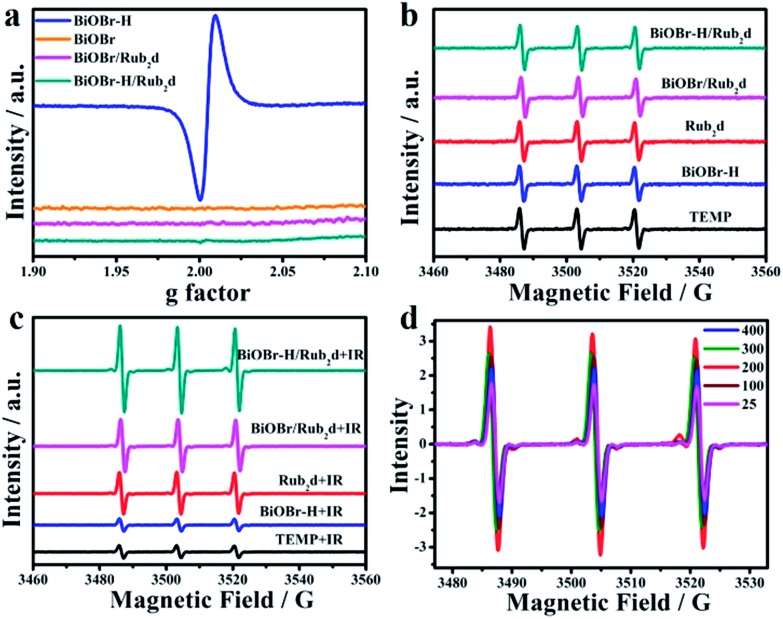
(a) ESR spectra of BiOBr–H, BiOBr, Rub_2_d and BiOBr–H/Rub_2_d (500 μg mL^–1^) without irradiation and (b) ESR spectra of BiOBr–H, BiOBr, BiOBr/Rub_2_d and BiOBr–H/Rub_2_d (500 μg mL^–1^) without irradiation and (c) with irradiation using a xenon lamp (100 mW cm^–2^) for 10 min. (d) ESR spectra of BiOBr–H/Rub_2_d with different loadings after irradiation for 10 min (Rub_2_d concentration 500 μg mL^–1^).

Electron Spin Resonance (ESR) was applied to quantify vacancy-induced ^1^O_2_ generation. As shown in Fig. 2a, the pristine BiOBr possessed few vacancies. However, the ESR spectrum of BiOBr–H revealed a greatly enhanced OV signal, resulting from the calcination step used in its synthesis. In comparison, BiOBr–H/Rub_2_d contained relatively few vacancies (as did BiOBr/Rub_2_d). Using 2,2,6,6-tetramethylpiperidine (Temp) as an ^1^O_2_ trap (Fig. 2b), BiOBr, BiOBr–H, BiOBr/Rub_2_d, BiOBr–H/Rub_2_d, and Rub_2_d all gave similar signals prior to irradiation. Following irradiation, the ^1^O_2_ signal of BiOBr–H/Rub_2_d increased significantly, whereas the signals of BiOBr/Rub_2_d and Rub_2_d did not change much. The ^1^O_2_ signal of irradiated BiOBr–H/Rub_2_d was twice that of Rub_2_d (Fig. 2c). Clearly, the abundant OVs in BiOBr–H/Rub_2_d are effective for the capture of electrons,^23^,^33^ thereby suppressing electron–hole pair recombination in the semiconductor. Accordingly, the excited electrons transferred from Rub_2_d under xenon lamp irradiation are captured by the OVs in BiOBr–H, leading to enhanced generation of ^1^O_2_.

To determine the optimal Rub_2_d loading capacity, a series of BiOBr–H/Rub_2_d (*x*%) were prepared and their ESR spectra were collected under xenon lamp irradiation (Fig. 2d). The ESR signal increased from BiOBr–H/Rub_2_d (25%) to BiOBr–H/Rub_2_d (200%). The signal intensity gradually decreased as the loading was increased from BiOBr–H/Rub_2_d (200%) to BiOBr–H/Rub_2_d (400%) (Fig. 2d and S15†). Therefore, BiOBr/Rub_2_d (200%) displayed the optimum performance and we used this Rub_2_d loading in all subsequent experiments. In addition, to further realize this oxygen-vacancy engineering mechanism, we further prove the generation of other radical species, *e.g.*, ·O_2_^–^ and ·OH of ROS. As expected, the ESR signal of ·O_2_^–^ and ·OH was also significantly enhanced as the irradiation time increased from 0 min to 10 min (Fig. S16†). This can further support our oxygen-vacancy engineering generation of ROS, which can be applied as a tool for the improvement of cancer therapy.

**Fig. 3:**
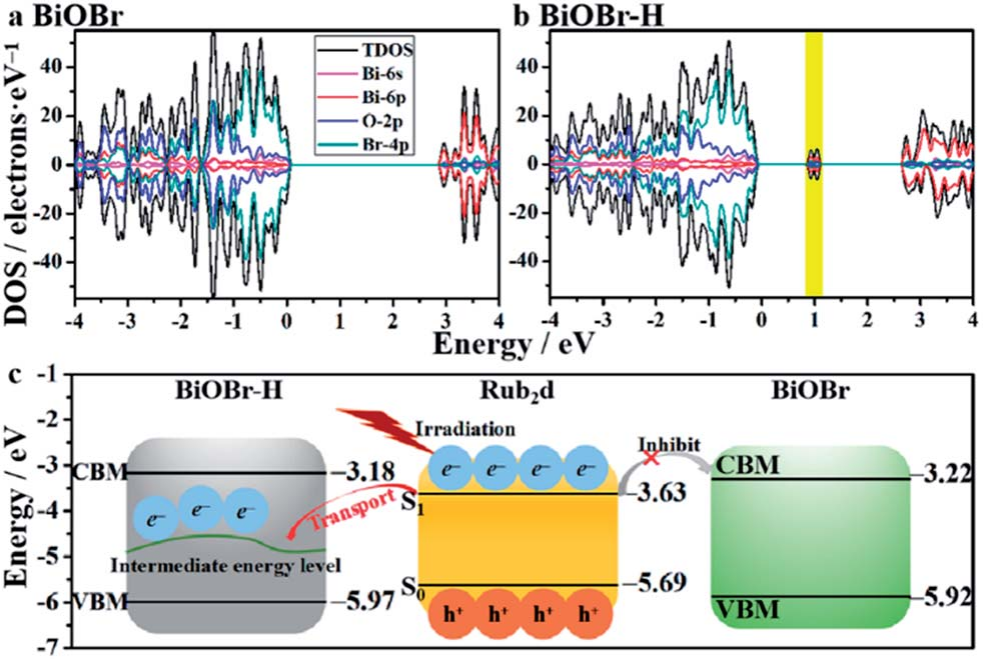
Band structures of BiOBr (a) and BiOBr–H (b). The band edge positions of BiOBr, BiOBr–H and Rub_2_d with the vacuum level set at 0 eV (c).

DFT calculations were used to gain deeper insights about the impact of oxygen-vacancy engineering on the generation of singlet oxygen. The schematic illustration of the structures of BiOBr and BiOBr–H is displayed in Fig. S17.† The surface energies of the low-index facets of BiOBr were first calculated and are listed in Table S3.† It was found that the (011) facet of BiOBr possesses the smallest surface energy (0.428 J m^–2^), revealing that the (011) facet will be preferentially exposed on the surface of BiOBr and BiOBr–H, in good agreement with the HRTEM findings (Fig. 1b). The density of states of both BiOBr and BiOBr–H is subsequently calculated and displayed in Fig. 3a and b. The band gap energy of BiOBr was calculated to be 2.70 eV, in good accord with that estimated from the UV-vis absorbance spectrum (Fig. S4†). For BiOBr–H, an intermediate energy level appears in the forbidden zone, resulting from the presence of abundant oxygen vacancies.^34^,^35^ The density of states of BiOBr–H indicates that the intermediate energy level is mainly composed of Bi-6p orbitals (Fig. S18†). Using the work functions determined for BiOBr and BiOBr–H, the positions of their conduction band minimum (CBM) and valence band maximum (VBM) were calculated (Fig. 3c). The HOMO and LUMO positions of the Rub_2_d complex were also calculated and are displayed in Fig. 3c. The calculations reveal that the photogenerated electrons in Rub_2_d can be efficiently transferred to the intermediate energy level of BiOBr–H with a large driving force of 1.311 eV, thereby facilitating rapid electron–hole separation. Similar defect-induced charge separation has also been reported in previous work.^36^,^37^ Furthermore, the binding energy between BiOBr–H and Rub_2_d was determined to be –4.482 eV, confirming the strong interaction between BiOBr–H and Rub_2_d.

### Measurements of singlet oxygen

We further investigated ^1^O_2_ generation *via* a chemical trapping experiment using 1,3-diphenylisobenzofuran (DPBF) as a trapping agent and Rose Bengal (RB) as a standard photosensitizer (^1^O_2_ quantum yield *Φ*_RB_ = 0.86 in ethanol). The absorption of DPBF at 410 nm was monitored under 520 nm irradiation, to follow the decay rate of the photosensitizing process. The detailed calculations used in this analysis are provided in Fig. S19.† The absorbance of the DPBF solution at 410 nm decreased quite quickly with irradiation time in the presence of BiOBr–H/Rub_2_d, confirming the fast generation of ^1^O_2_. In comparison, the decrease in the absorbance at 410 nm was relatively slow in the presence of BiOBr/Rub_2_d and Rub_2_d, again confirming that the OVs in BiOBr–H/Rub_2_d facilitate ^1^O_2_ generation. The ^1^O_2_ quantum yield of BiOBr–H/Rub_2_d was calculated to be 0.49, which is approximately 2-fold higher than that of Rub_2_d alone (0.22). To further evaluate the benefits of the oxygen vacancies in BiOBr–H for promoting ^1^O_2_ generation, we loaded the photosensitizers zinc phthalocyanine (ZnPc) and indocyanine green (ICG) on BiOBr–H and studied the generation of ^1^O_2_*via* UV-vis spectroscopy. The singlet oxygen yields of BiOBr–H/ZnPc and BiOBr–H/ICG were 0.50 and 0.28, respectively (Table S4†), much higher than those of ZnPc (0.31) and ICG (0.15) (Fig. S20 and S21†). Photocurrent response measurements provided further evidence for the excellent charge transfer properties of BiOBr–H/Rub_2_d. As expected, Rub_2_d and BiOBr–H showed only a weak photocurrent response, whereas BiOBr–H/Rub_2_d showed a remarkable photocurrent response (Fig. S22†). Therefore, it can be concluded that the incorporation of the probe Rub_2_d into BiOBr–H can accelerate the generation of ^1^O_2_ from the Rub_2_d. To summarize, adsorption of Rub_2_d on OV-rich BiOBr–H enhances the population of the Rub_2_d triplet state (evidenced by a phosphorescence lifetime increase). Facile electron transfer occurs from excited Rub_2_d to BiOBr–H.

### *In vitro* anticancer activity

The internalization and *in vitro* cellular bio-imaging of the BiOBr–H/Rub_2_d was assessed on the Hela cell line using a Laser Scanning Confocal Microscope (LSCM) with an excitation wavelength of 488 nm. After incubation with BiOBr–H/Rub_2_d for 24 h, the nucleus of the cells was stained with DAPI which gives a blue color emission. Fig. S23† shows that the BiOBr–H/Rub_2_d was internalized by cells through endocytosis^38^ and was mainly located in the cytoplasm. Furthermore, from the TEM observation (Fig. S24†), the BiOBr–H/Rub_2_d samples can be internalized by the Hela cells and then enter mitochondria while the others accumulate in lysosomes.^38^ In addition, elemental mapping can vividly monitor the Bi, Br and Ru distribution during the cell culture and the results confirmed the stability of BiOBr–H/Rub_2_d inside Hela cells (Fig. S25†). We further investigated the two-photon imaging properties of BiOBr–H/Rub_2_d using three various cell lines: Hela, HepG-2 and MCF-7 cells. As shown in Fig. S26,† the cells exhibit fluorescence in the green and red channels under 800 nm excitation. This confirms the potential of BiOBr–H/Rub_2_d as an imaging agent for the detection of cancer cells *via* two-photon fluorescence imaging techniques.

**Fig. 4:**
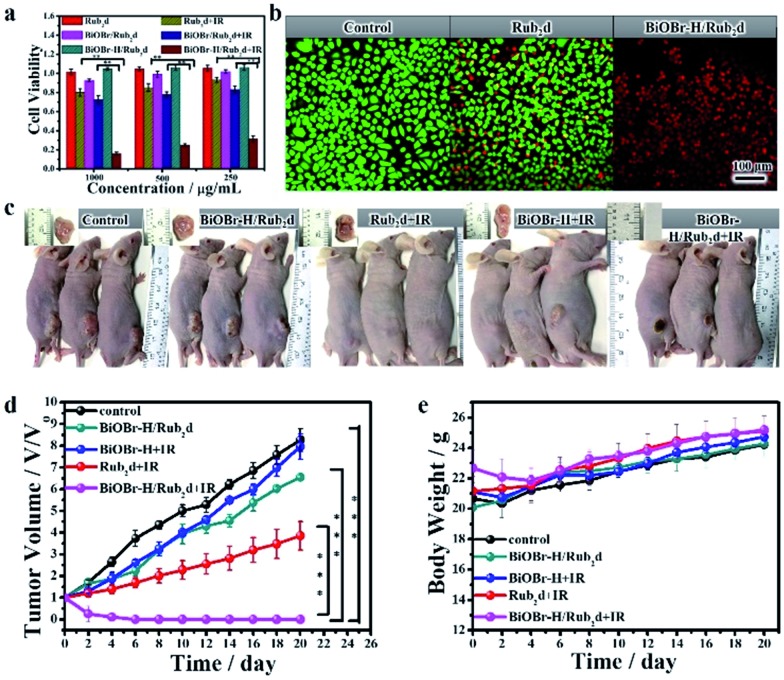
(a) Cell viability of Hela cells incubated with Rub_2_d, BiOBr–H/Rub_2_d or BiOBr/Rub_2_d for 24 h with and without irradiation (xenon lamp, 100 mW cm^–2^), respectively. The viability was the average of six measurements (*n* = 6). (b) Fluorescence imaging of Rub_2_d and BiOBr–H/Rub_2_d with and without IR therapy, respectively. Live/dead Hela cells are green/red (calcein AM/PI), respectively. (c) *In vivo* photographs of various groups of mice administrated with 200 μL of PBS, BiOBr–H, Rub_2_d or BiOBr–H/Rub_2_d with or without 520 nm irradiation for 10 min (xenon lamp, 100 mW cm^–2^). (d) Tumor size and (e) body weight. The tumor volume was the average of five measurements (*n* = 5) and the error bars indicate the standard deviation. *p < 0.05, **p < 0.01, and ***p < 0.001.

To confirm the anticancer activity of BiOBr–H/Rub_2_d was solely due to the photodynamic therapy, the cytotoxicity of BiOBr and BiOBr–H was checked by the CCK8 assay in Hela cells (Fig. S27†). After 24 h of incubation, BiOBr and BiOBr–H showed negligible toxicity towards the Hela cells even at concentrations up to 500 μg mL^–1^. Further, Rub_2_d, BiOBr/Rub_2_d, and BiOBr–H/Rub_2_d also showed negligible toxicity (Fig. 4a). Rub_2_d and BiOBr/Rub_2_d slightly inhibited the growth of the Hela cells, following irradiation for 10 min (Fig. 4a). Remarkably, the growth of Hela cells was significantly inhibited when incubated with BiOBr–H/Rub_2_d and irradiated at 520 nm (100 mW cm^–2^) for 10 min. Similar results were obtained for the MCF-7 and HepG-2 cell lines (Fig. S28 and S29†). To visualize the PDT effect on the cells, the dead and live cells were stained with Propidium Iodide (PI) and calcein-AM, respectively (Fig. 4b). It is apparent that the majority of cells were dead after being treated with the BiOBr–H/Rub_2_d and irradiated, whereas they remained in relatively good condition without irradiation. The effect of illumination on Rub_2_d treated cells is less obvious. Again, the data point to a synergistic effect resulting from the adsorption of Rub_2_d on BiOBr–H. To validate the benefits of OV-engineering for ROS generation *in vitro*, 2′,7′-dichlorofluorescin diacetate (DCFH-DA) was used. Following diffusion into cells, DCFH-DA is deacetylated by cellular esterases to give a non-fluorescent compound, which can be oxidized by ROS into 2′,7′-dichlorofluorescein (DCF), which has characteristic excitation and emission maxima of 488 and 525 nm, respectively.^39^ Hela cells treated with phosphate-buffered saline (PBS) and irradiated at 520 nm with a luminous power of 100 mW cm^–2^ for 10 min showed a typical Hela cell green fluorescence signal (Fig. S30†). Cells incubated with BiOBr–H/Rub_2_d and Rub_2_d showed a significantly enhanced green fluorescence due to the generation of DCF. The intensity of the fluorescence signal was strongly dependent on the incubation time. However, at all incubation times BiOBr–H/Rub_2_d demonstrated the strongest green fluorescence signal.

### Fluorescence imaging and *in vivo* antitumor assay

Encouraged by the *in vitro* PDT efficacy of BiOBr–H/Rub_2_d, *in vivo* PDT experiments were subsequently performed involving monitoring of the volume of the tumors after irradiation. Hela tumor-bearing mice were divided into 5 groups (control group, BiOBr–H/Rub_2_d, BiOBr + IR, Rub_2_d + IR, and BiOBr–H/Rub_2_d + IR), with each group having 5 mice (Fig. 4c). No significant therapeutic effect was seen for the BiOBr–H/Rub_2_d group without irradiation and BiOBr with irradiation. In contrast, the tumor volumes for the Rub_2_d + IR group decreased. Notably, the tumor volume of the BiOBr–H/Rub_2_d + IR group was dramatically reduced compared with that of the Rub_2_d + IR group (Fig. 4d), demonstrating that the high local concentration of ROS generated by BiOBr–H/Rub_2_d under irradiation greatly improved the effectiveness of PDT therapeutic effects. In addition, no apparent body weight losses were found during the whole treatment (Fig. 4e). Imaging studies on the excised major organs and the tumor showed the strong fluorescence of the isolated tumor tissue after the i.v. injection of BiOBr–H/Rub_2_d for 10 min and 24 h, respectively (Fig. S31 and S32†). To further investigate the therapeutic effect of each treatment group, the tumor tissues were analysed by hematoxylin and eosin (H&E) staining after the treatment. As shown in Fig. S33,† the main organs including the heart, liver, spleen, lungs and kidneys showed no obvious damage after BiOBr–H/Rub_2_d + IR treatment, while there was serious necrosis in the tumor tissues after the treatment. These results demonstrate the negligible toxicity of the BiOBr–H/Rub_2_d PDT treatment and excellent therapeutic potential of BiOBr–H/Rub_2_d for cancer therapy.

## Conclusions

In summary, under the hypothesis that abundant OVs in BiOBr–H enable the improvement of the photocatalytic activity of a photosensitizer, we developed a novel theranostic agent by functionalizing the BiOBr–H with a PS (Rub_2_d). The resultant BiOBr–H/Rub_2_d indeed demonstrates outstanding therapeutic properties for PDT, which originated from the efficacy of OVs in the enhancement of ROS generation in tumor cells during light irradiation, conferring strong ablation effects both *in vitro* and *in vivo*. The phenomenon can be explained using density functional theory (DFT) calculations. All results suggest the feasibility of the concept (OVs can enhance ROS generation). Therefore, this work highlights the potential of vacancy engineering in the development of effective theranostic agents for cancer therapy.

## Conflicts of interest

There are no conflicts to declare.

## Supplementary Material

Supplementary information
