# Supplementary material for: Vacancy-enhanced generation of singlet oxygen for photodynamic therapy
Source: Chem Sci. 2018 Dec 20;10(8):2336–41. doi: 10.1039/c8sc05275a (PMC6385666; doi:10.1039/c8sc05275a)
Supplement: Supplementary file 1 [file SC-010-C8SC05275A-s001.pdf]

Electronic Supplementary Material (ESI) for Chemical Science.
This journal is © The Royal Society of Chemistry 2018

Electronic Supplementary Information

Vacancy-enhanced Generation of Singlet Oxygen for Photodynamic Therapy

Shanyue Guan,[†a] Li Wang,[†a, b] Si-Min Xu,[†c] Di Yang,[b] Geoffrey I.N. Waterhouse,[d] Xiaozhong Qu,*[b] and Shuyun Zhou*[a]

[a] *Key Laboratory of Photochemical Conversion and Optoelectronic Materials, Technical Institute of Physics and Chemistry, Chinese Academy of Sciences, Beijing, 100190, China.*

[b] *University of Chinese Academy of Sciences, Beijing 100190, China.*

[c] *State Key Laboratory of Chemical Resource Engineering, Beijing University of Chemical Technology, 100029, Beijing, P. R. China*

[d] *School of Chemical Sciences, The University of Auckland, Auckland 1142, New Zealand.*

* Corresponding authors.

E-mail addresses: zhou_shuyun@mail.ipc.ac.cn (S.Z); quxz@iccas.ac.cn (X.Q.).

[†]These authors equally contributed to this work.

Experimental Section

Reagents and Chemicals

Ruthenium (II) chloride, 2,2'-bipyridine, biisonicotinic acid, 4′,6-diamidine-2′-phenylindole dihydrochloride (DAPI), calcein-AM (Cal-AM) and propidium iodide (PI) were purchased from Sigma-Aldrich Corporation. Analytical grade chemicals including $Bi(NO_3)_2 \cdot 5H_2O$, KBr, polyvinylpyrrolidone and ethylene glycol were obtained from Sinopharm Chemical Reagent Co., Ltd. Dulbecco's modified eagle's medium (DMEM), fetal bovin serum (FBS) and phosphate buffer solution (PBS) were obtained from Beijing Solarbio Science and Technology Co., Ltd. Cell counting kit (CCK-8) was purchased from Dojindo China Co., Ltd. DI water was used in all experiments.

Synthesis of $Ru(bpy)_2C\text{-}pyCl_2$

Ruthenium chloride (0.05 g, 0.24 mmol) and 2,2'-bipyridine (0.075 g, 0.48 mmol) were dissolved in 25 mL of anhydrous *N, N*–dimethylformamide (DMF), and refluxed at 120 °C for 12 h under a nitrogen atmosphere. The reaction liquid was filtered and the filtrate evaporated to dryness under vacuum. The obtained $Ru(bpy)_2Cl_2$ was washed three times with diethyl ether, collected by centrifugation at 8000 r.p.m and then allowed to dry naturally at room temperature. The product (0.02 g, 0.04 mmol) was then dissolved in anhydrous DMF, to which 2,2'-biisonicotinic acid (0.010 g, 0.04 mmol) was added and the resulting mixture stirred at 120 °C for 12 h in the dark under a nitrogen atmosphere. The $Ru(bpy)_2C\text{-}pyCl_2$ product was collected by centrifugation, washed several times with diethyl ether, then air dried. For simplicity, $Ru(bpy)_2C\text{-}pyCl_2$ is denoted as Rub_2d in this manuscript.

Synthesis of BiOBr with and without oxygen vacancies (OVs)

For the preparation of BiOBr without oxygen vacancies, the following procedure was used. $Bi(NO_3) \cdot 5H_2O$ (3 mmol) and 2 mg of polyvinylpyrrolidone (PVP) were added slowly to 32 mL of an ethylene glycol solution containing 3 mmol of KBr. The resulting

mixture was then stirred for 1 h at room temperature in air, then poured into a 100 mL Teflon-lined stainless autoclave. The autoclave was then heated at 160 °C for 12 h under autogenous pressure, and then allowed to cool naturally to room temperature. The solid precipitate was collected and washed several times with deionized water and then ethanol. The product was then dried at 60 °C in vacuum. BiOBr with OVs, denoted herein as BiOBr-H, was obtained by heating BiOBr at 300 °C in an O_2 atmosphere for 4 h.

Synthesis of BiOBr-H/Rub$_2$d

A solution of Rub$_2$d in DI water (1 mg/mL) was added to a suspension of BiOBr-H in DI water (BiOBr-H concentration ranging from 0.25–4 mg mL^{-1}) under magnetic stirring, and the stirring continued for 20 min at RT. The BiOBr-H/Rub$_2$du products were then collected by centrifugation and re-dispersed in water for later use.

Synthesis of BiOBr-H/PS

A solution of photosensitizers (denoted as PS, e.g. zinc phthalocyanine and idocyanine green) in DI water (1 mg/mL) was added to a suspension of BiOBr-H in DI water (1 mg /mL) under magnetic stirring, and the stirring continued for 20 min at RT. The BiOBr-H/PS products were then collected by centrifugation and re-dispersed in water for later use.

Model construction

The model of bulk BiOBr was constructed in the space group of P4/nmm with the following lattice parameters: $a = b = 3.92$ Å, $c = 8.11$ Å, $a = \beta = \gamma = 90°$.[1] The supercell was 2 × 2 × 2 in the *a*-, *b*-, and *c*- directions. Therefore, the chemical formula of the model is $Bi_{16}O_{16}Br_{16}$. The model of the defected BiOBr-H was built by removing one O atom from the model of BiOBr. Thus the model of BiOBr-H possessed the chemical formula of $Bi_{16}O_{15}Br_{16}$. The model for Rub$_2$d was constructed according to its molecular formula (Figure S1). To study the surface energies of different exposed facets for BiOBr, 7 kinds of low-index facets were built ((001), (010), (100), (011), (101), (110), and (111)). These facets all possessed the same formula unit as bulk BiOBr (i.e.,

$Bi_{16}O_{16}Br_{16}$) and 15 Å of vacuum layer. The (011) facet was determined to be the preferentially exposed surface and thus used in subsequent calculations.

The model of BiOBr/Rub_2d was constructed by placing one Rub_2d molecule on the (011) facet of BiOBr. The model of BiOBr-H/Rub_2d was constructed in the same way by placing one Rub_2d molecule on the (011) facet of BiOBr-H.

Computational methods

All the calculations were performed using the $DMol^3$ code in the Materials Studio 5.5 software package (BIOVIA Corp.).[2] The generalized gradient approximation (GGA) Perdew-Burke-Ernzerhof (PBE) functional[3] and double numerical plus polarization version 4.4 basis set[4] were applied. Reciprocal-space integration over the Brillouin zone is approximated through Γ-point sampling (3 × 3 × 3 Monkhorst-Pack grid). A density mixing fraction of 0.2 with direct inversion in the iterative subspace was employed. The convergence tolerances of energy, gradient, and displacement were 1.0×10^{-5} hartree, 2.0×10^{-3} hartree/Å, and 5.0×10^{-3} Å, respectively. The band structures of BiOBr and BiOBr-H, together with the HOMO and LUMO of Rub_2d, were calculated.

In order to determine the most preferentially exposed facets of BiOBr, the surface energy (γ) of BiOBr was derived from equation 1:[5]

$$\gamma = \frac{E_{\text{slab}} - E_{\text{bulk}}}{2A} \quad (1)$$

where E_{slab} is the total energy of the optimized slab possessing the same formula unit as bulk BiOBr, E_{bulk} is the energy of bulk BiOBr, and A is the surface area for one side of the slab.

The electronic band gap energy, E_g, of BiOBr was calculated using equation 2:[6]

$$E_g = E_{\text{CBM}} - E_{\text{VBM}} \quad (2)$$

where E_{CBM} and E_{VBM} represent the energy of the conduction band minimum (CBM) and valence band maximum (VBM), respectively. By calculating the band structure of BiOBr or BiOBr-H, the energy difference (x) between the Fermi level (E_F) and the CBM can be obtained by equation 3:

$$x = E_{\mathrm{CBM}} - E_{\mathrm{F}} \quad (3)$$

The work function of the (011) facet of BiOBr can be calculated using equation 4:

$$W = -e\phi - E_{\mathrm{F}} \quad (4)$$

where e is the charge of an electron, ϕ is the electrostatic potential in the vacuum close to the surface. The values of ϕ and E_F can be directly obtained from the $DMol^3$ code.

The band edge positions of BiOBr and BiOBr-H were then calculated using equations 5 and 6:[7]

$$E_{\mathrm{CBM}} = E_{\mathrm{F}} + x = -W + x \quad (5)$$

$$E_{\mathrm{VBM}} = E_{\mathrm{CBM}} - E_{\mathrm{g}} = -W + x - E_{\mathrm{g}} \quad (6)$$

The positions of the HOMO and LUMO for Rub_2d were directly obtained from the $DMol^3$ code by analyzing the molecular orbitals.

The binding energy, E_{bind}, between Rub_2d and BiOBr was calculated with equation 7:[8]

$$E_{bind} = E_{BiOBr/Rub_2d} - E_{BiOBr} - E_{Rub_2d} \quad (7)$$

Where E_{BiOBr/Rub_2d}, E_{BiOBr}, and E_{Rub_2d} is the energy of BiOBr/Rub_2d, BiOBr, and Rub_2d, respectively. E_{bind} between Rub_2d and BiOBr-H was calculated *via* a similar method.

In vitro experiments

The *in vitro* cytotoxicity of various samples were evaluated on human various cells (Hela, MCF-7, HepG-2). Specifically, human various cells were incubated in a 25 cm^2 cell-culture flask and then the cells (1×10^4 cells/well) were seeded into a 96-well plate, respectively. After seeding, the Hela cells were exposed to a series doses of composites for 24 h. After further incubation for 24 h, a mixture of CCK-8 and DMEM (1:10) was added to each well of the 96-well plate. The cell viability was calculated as the ratio of the absorbance of the wells. The absorbance at 450 nm was measured using Biotek synergy H_1(USA) multi-mode microplate reader. The cytotoxicity was calculated as the average of six individual reads from six wells.

***In vivo* experiments**

All animal procedures were performed in accordance with the Guidelines for Care and Use of Laboratory Animals of Technical Institute of Physics and Chemistry, Chinese Academy of Sciences and Experiments were approved by the Animal Ethics Committee of Technical Institute of Physics and Chemistry, Chinese Academy of Sciences. Male nude Balb/c mice were purchased from Beijing HFK Bioscience Co., Ltd. 2×10^6 Hela cells suspended in 200 μL DMEM were injected subcutaneously into the right lateral back of each mouse. Mice bearing Hela tumors were treated when the tumor volume reached ~100 mm^3. The mice were randomized into 5 groups. Two groups of animals (each n = 5) were dosed with PBS or BiOBr-H/Rub_2d at 10 mg/kg in 200 μL of saline (no irradiation control groups). The other 3 groups of mice (n = 5) were dosed with 200 μL of BiOBr-H, Rub_2d or BiOBr-H/Rub_2d at 10 mg/kg *via* intravenous injection with irradiation. Ten minutes after injection, the tumor sites were irradiated with visible light for 10 min. This visible light source was obtained by a 300 W Xenon lamp (EOSun, Au-Light, America) with cutoff filter (520 ± 15 nm). The irradiance was measured to be 100 mW/cm^2 (CEL-NP2000). After that, the tumor size and the body weight of the animals were monitored every two days. No further light treatments were performed. The volume of the tumor was calculated using the equation of $V = (L\times W^2)/2$, where L and W are the length and width of the tumor, respectively, measured using a caliper. Twenty days after the therapy, the mice were euthanized and the main organs and tumor dissected and fixed in a 4% formaldehyde solution for 24 h at room temperature. The slices of the organs were stained with hematoxylin and eosin and investigated for histological variations.

Measurement of singlet oxygen quantum yield.

The singlet oxygen ($^1O_2$) quantum yields of Rub_2d, BiOBr/Rub_2d, BiOBr-H/Rub_2d, ICG, BiOBr/ICG, BiOBr-H/ICG, ZnPc, BiOBr/ZnPc, and BiOBr-H/ZnPc were obtained using a previously reported method[9]. In order to calculate the singlet oxygen yield, 1,3-diphenylisobenzofuran (DPBF) was used as the capture agent and Rose Bengal (RB) as a reference. A Xenon lamp of power of 100 mW/cm^2 with monochromatic light filters (520 ± 15 nm, 600± 15 nm, 800 ± 15 nm) was used as the

light source. The yield of singlet oxygen is positively correlated with the rate of decrease of the DPBF absorption signal at 410 nm. Accordingly, the absorbance of DPBF in the solution at 410 nm was monitored as a function of irradiation time. Singlet oxygen yields were determined from plots of $A_{410\ nm}$ versus time by assuming first-order kinetics and using Equation (9):

$$\emptyset_S = \emptyset_{RB} \frac{K_S * A_{RB}}{K_{RB} * A_S} \quad (9)$$

Where $\emptyset_S$ represents the singlet oxygen ($^1O_2$) quantum yield of samples, $\emptyset_{RB}$ equals 0.86 for the singlet oxygen ($^1O_2$) quantum yield of RB in the ethanol, K_{RB} and K_S represent rate constants for the disappearance of DPBF in RB and sample solutions, respectively. A_S and A_{RB} are the light absorbed by the samples and RB, respectively.

Sample characterization

Two-photon imaging was performed on a ARsiMP-LSM-Kit-Legend Elite-USX confocal fluorescence instrument under 800 nm irradiation. Confocal fluorescence images were obtained on a Nikon A1R Eclipse Ti confocal laser scanning microscope with a 40× water immersion objective. X-ray diffraction (XRD) patterns were recorded on a D8 focus diffractometer (Bruker), using a Cu Kα source (λ = 0.15418 nm) operating at 40 kV and 30 mA. Zeta potentials were determined on a MALVERN 2000. Photoluminescence spectra were collected on a RF-5301PC fluorospectrophotometer using 488 nm excitation. UV-vis absorption spectra were collected in the range 200–900 nm on a Shimadzu U-3000 spectrophotometer, using a slit width of 1.0 nm. Fluorescence decay curves were obtained on an Edinburgh Instruments FLS980 fluorimeter. Average fluorescence lifetimes were calculated from the decay curves by fitting multiple-exponential functions. Transmission electron microscopy (TEM) images were recorded on a JEM-2100F high resolution transmission electron microscope operating at an accelerating voltage of 200 kV. Fourier transform infrared (FT-IR) spectra were obtained on a Varian Excalibur 3100 FTIR spectrometer over the range 4000-500 cm^{-1} at 2 cm^{-1} resolution. X-ray photoelectron spectra (XPS) were collected on a PHIQ2000 X-ray photoelectron spectrometer equipped with an Al Kα X-ray source. Electron spin resonance (ESR) spectra were collected on a Bruker 500 spectrometer. A Thermo Multiskan FC was used to investigate cell viability. The visible light source was obtained by a 300 W Xenon lamp (EOSun, Au-Light, America) with

cutoff filter (520 ± 15 nm). The irradiance was measured to be 100 mW/cm^2 (CEL-NP2000).

Statistical Analysis

Statistical significance was assessed using one-way ANOVA analyses on SPSS 16.0 software. The difference was considered to be statistically significant if the probability value was less than 0.05 (i.e. $p < 0.05$). Mean values and standard deviations (SD) were calculated from replicate experiments. Data were presented as mean ± SD.

Supplementary Figures

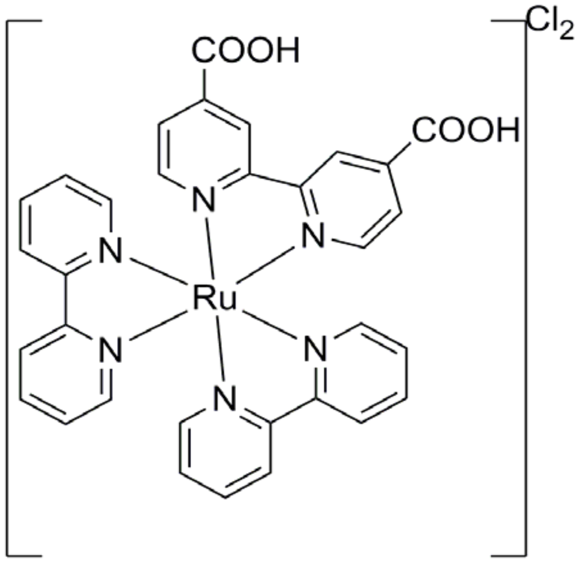

Figure S1. The chemical structure of Rub2d.

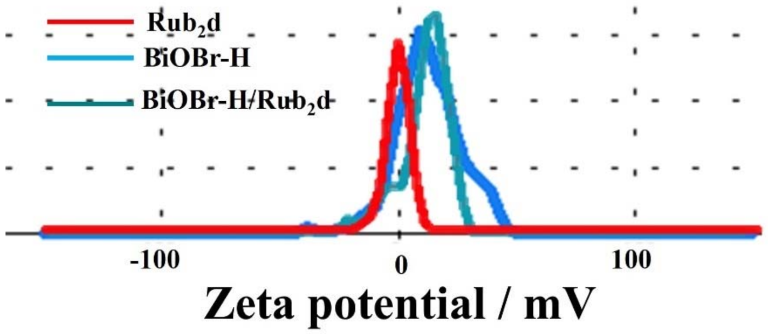

Figure S2. Zeta potential of BiOBr-H, Rub2d and BiOBr-H/Rub2d.

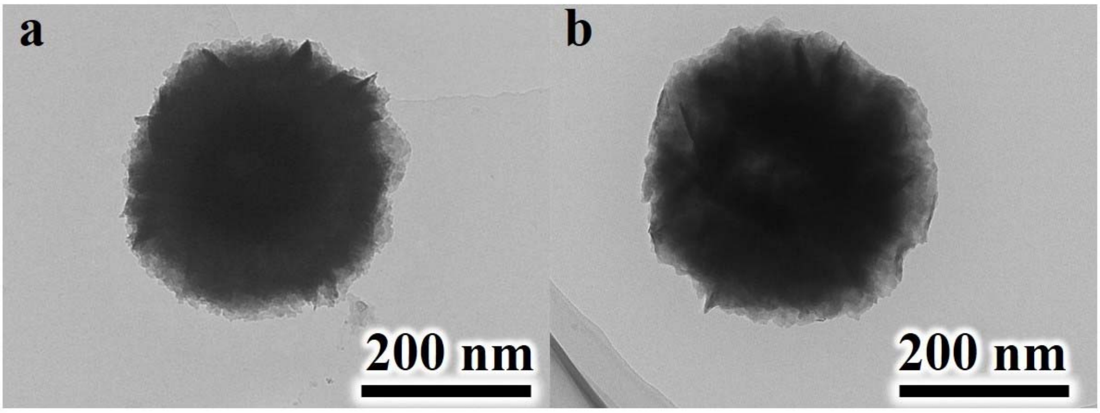

Figure 3. TEM images of BiOBr-H after incubation after incubation in the cell culture medium (a) or the buffers solution with pH=6.5 including H_2O_2 (tumor microenvironment) for 24 h (b).

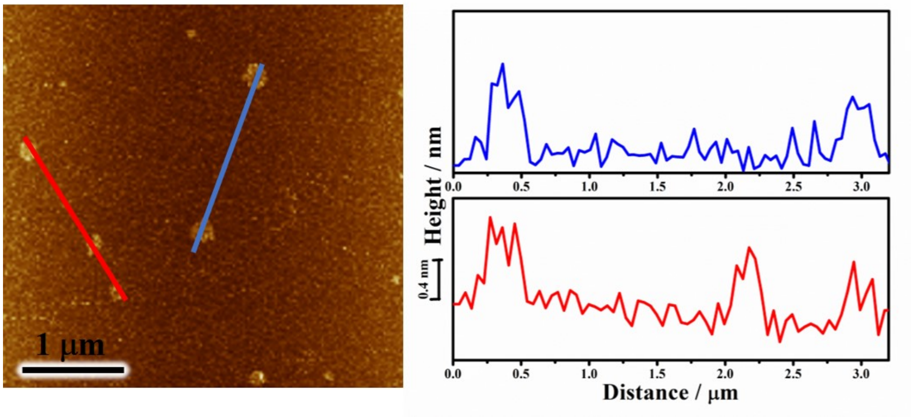

Figure S4. AFM images of BiOBr-H single layer.

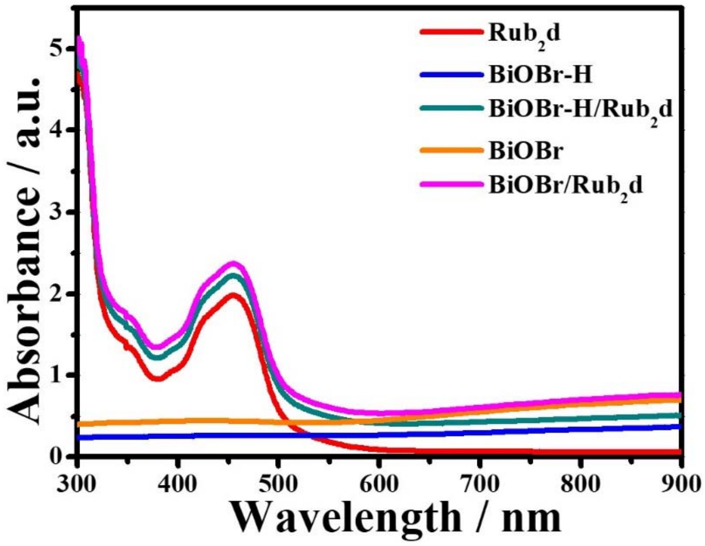

Figure S5. UV-vis spectra for BiOBr, BiOBr/Rub$_2$d, Rub$_2$d, BiOBr-H, and BiOBr-H/Rub$_2$d.

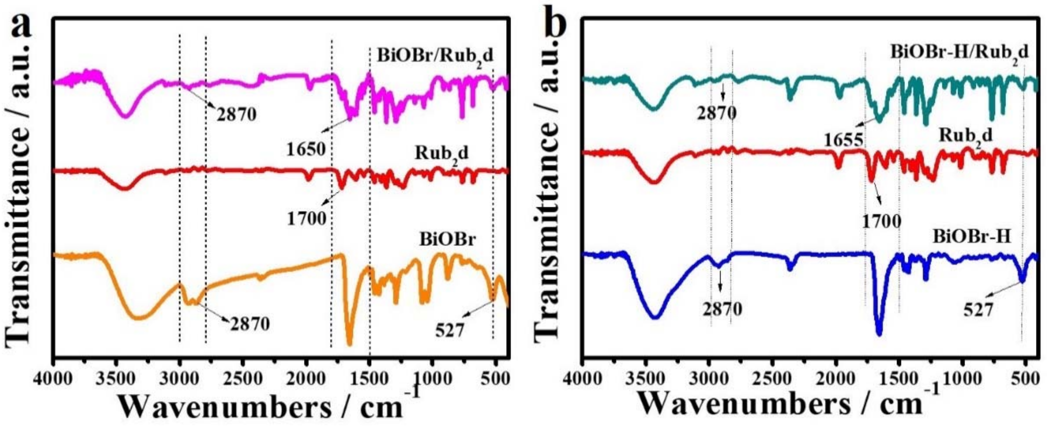

Figure S6. FT-IR spectra for (a) BiOBr, Rub$_2$d and BiOBr/Rub$_2$d, and (b)BiOBr-H, Rub$_2$d and BiOBr-H/Rub$_2$d.

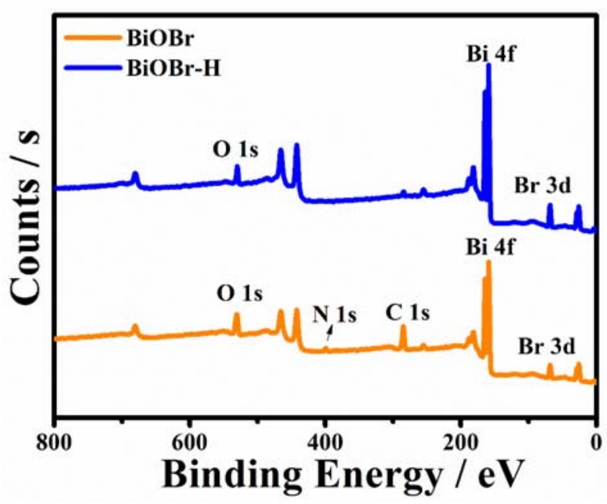

Figure S7. XPS survey spectra of BiOBr and BiOBr-H.

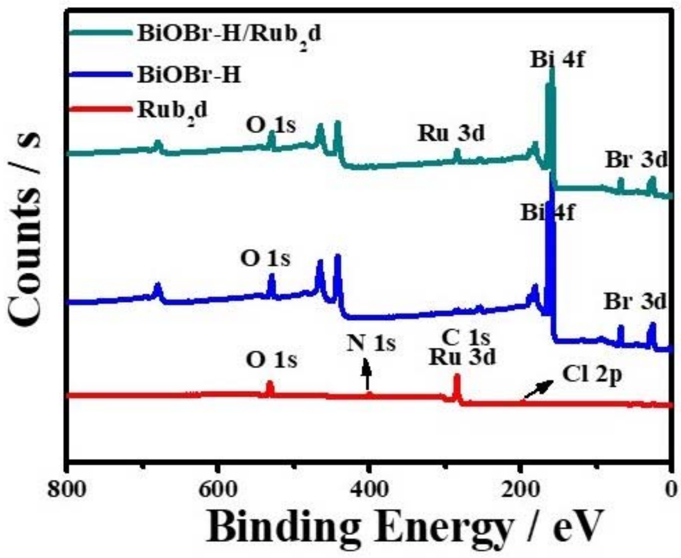

Figure S8. XPS survey spectra for Rub_2d, BiOBr-H and BiOBr-H/Rub_2d.

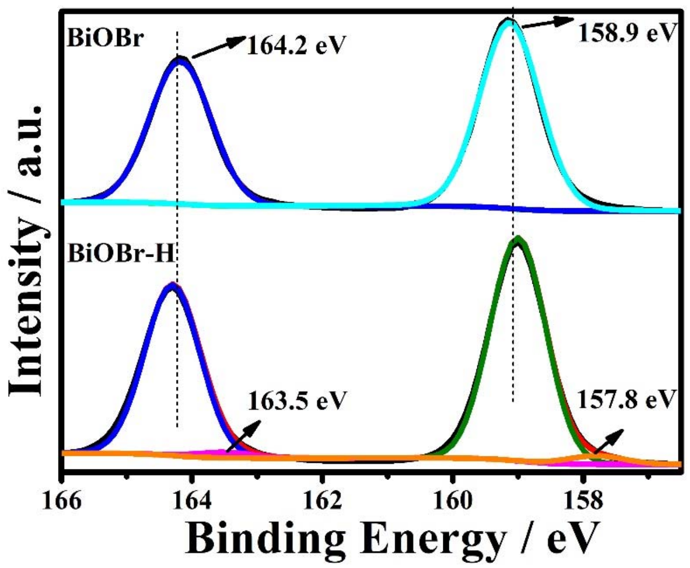

Figure S9. High-resolution Bi 4f XPS spectra for BiOBr and BiOBr-H.

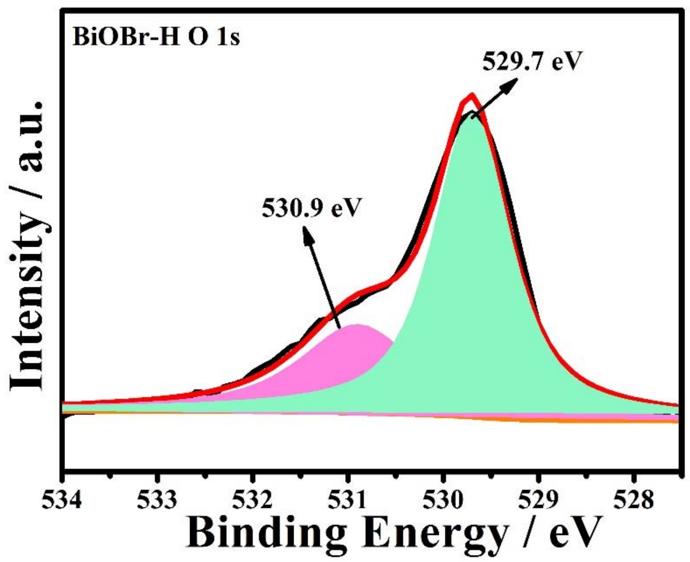

Figure S10. High-resolution O 1s XPS spectra for BiOBr-H.

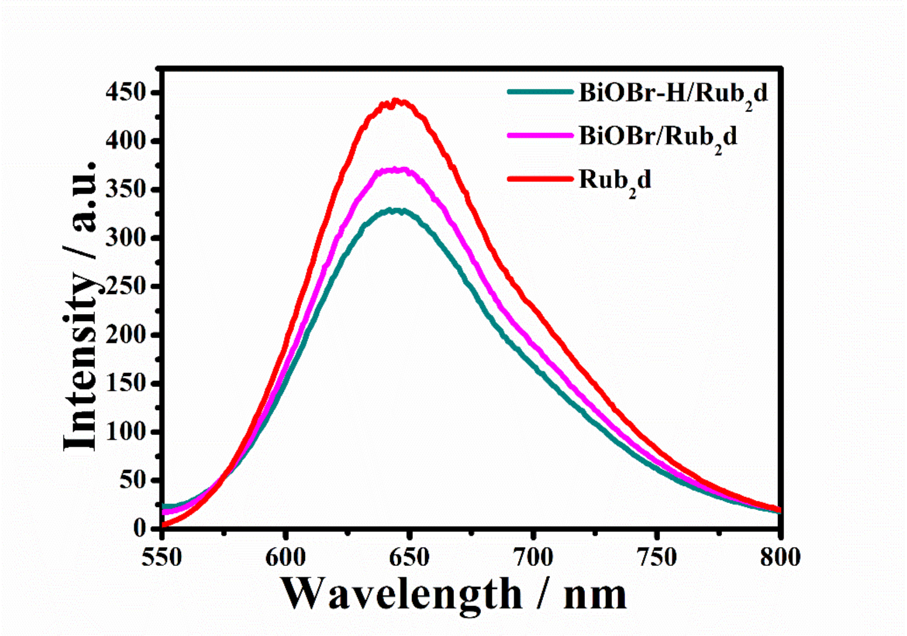

Figure S11. Fluorescence spectra for BiOBr/Rub2d, BiOBr-H/Rub2d and Rub2d under 488 nm excitation.

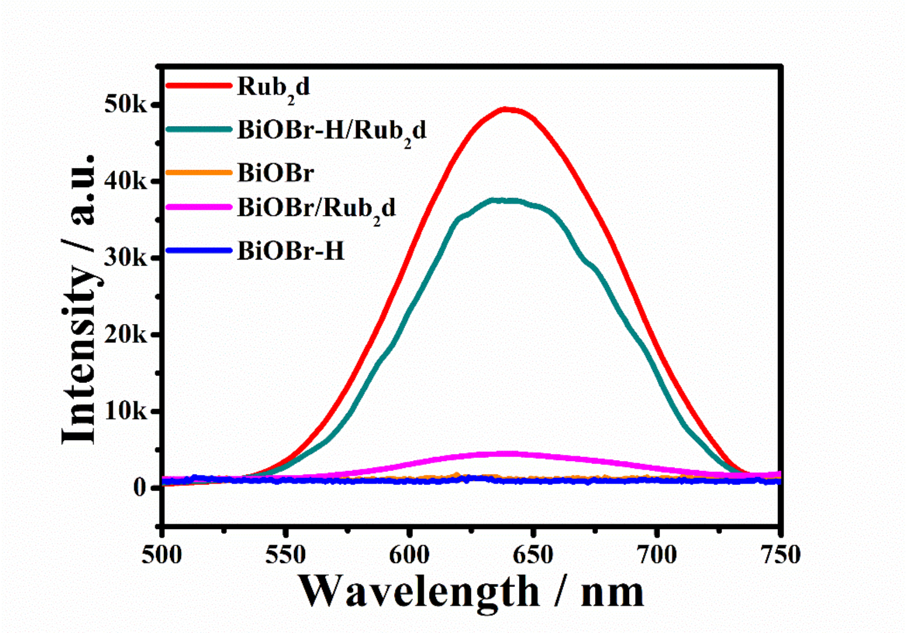

Figure S12. Two-photon fluorescence emission spectra for BiOBr-H, BiOBr, Rub2d, BiOBr/Rub2d and BiOBr-H/Rub2d under 800 nm excitation.

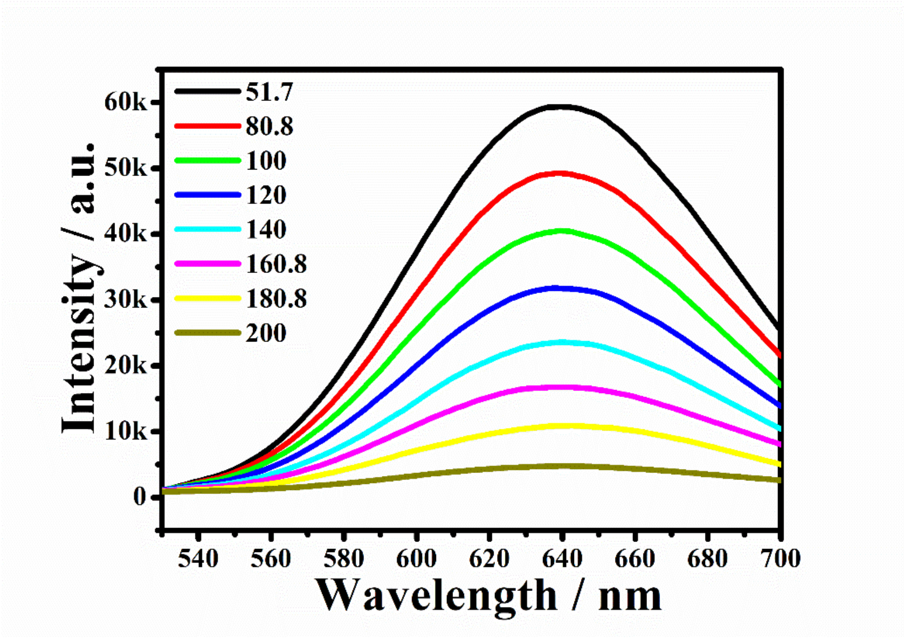

Figure S13. Two-photon fluorescence emission spectra for BiOBr-H/Rub2d under 800 nm excitation and different laser powers.

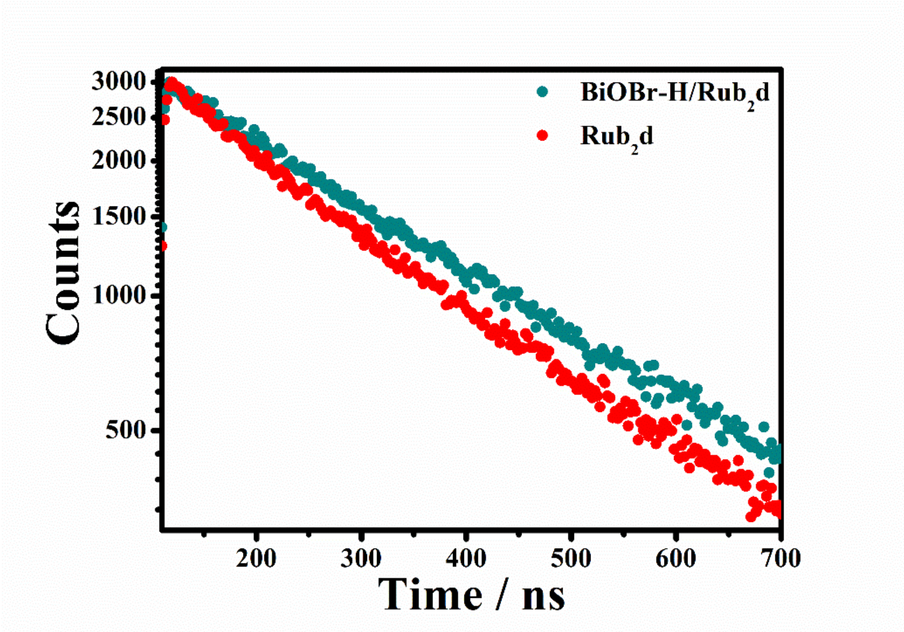

Figure S14. Fluorescence lifetime curves for Rub2d and BiOBr-H/Rub2d.

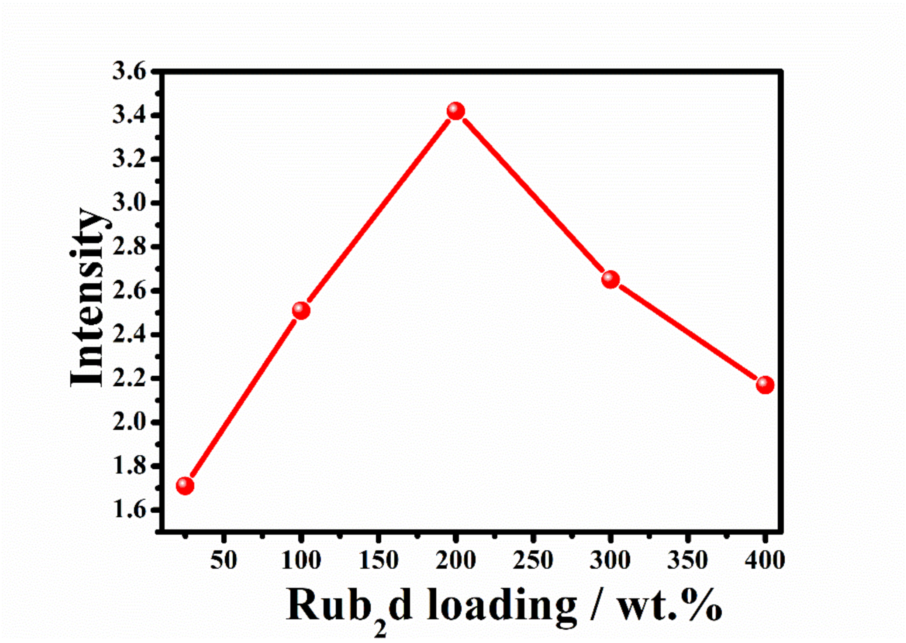

Figure S15. ESR signal intensities for BiOBr-H/Rub2d at different Rub2d loadings.

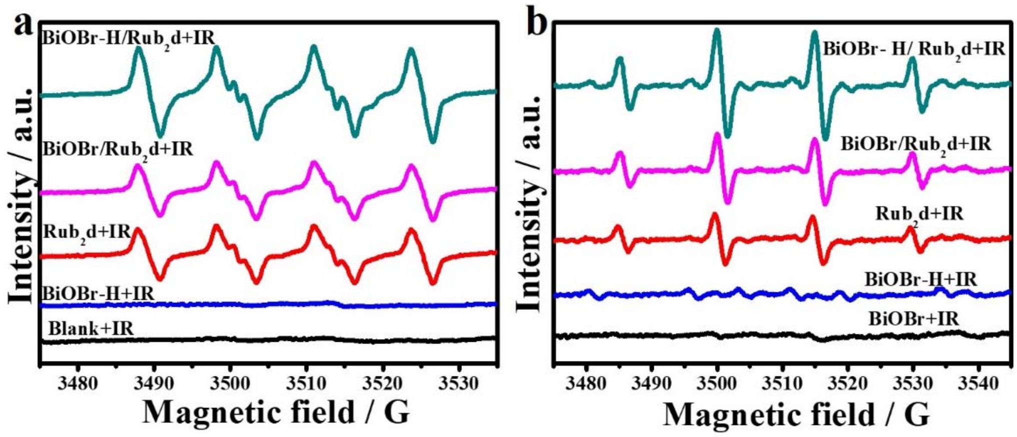

Figure S16. The ESR signals of superoxide radical ($\cdot O_2^-$) and hydroxyl radical ($\cdot OH$) for BiOBr-H, BiOBr, Rub2d, BiOBr/Rub2d and BiOBr-H/Rub2d (500 ug/mL), respectively under irradiation for 10 min.

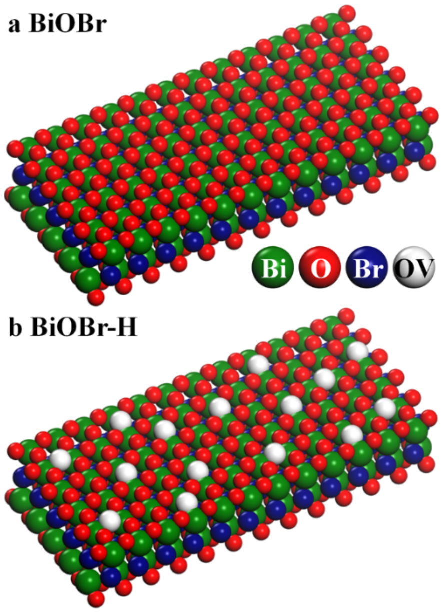

Figure S17. Schematic illustrations of the oxygen vacancy (OV)-free BiOBr, and oxygen vacancy-abundant BiOBr-H.

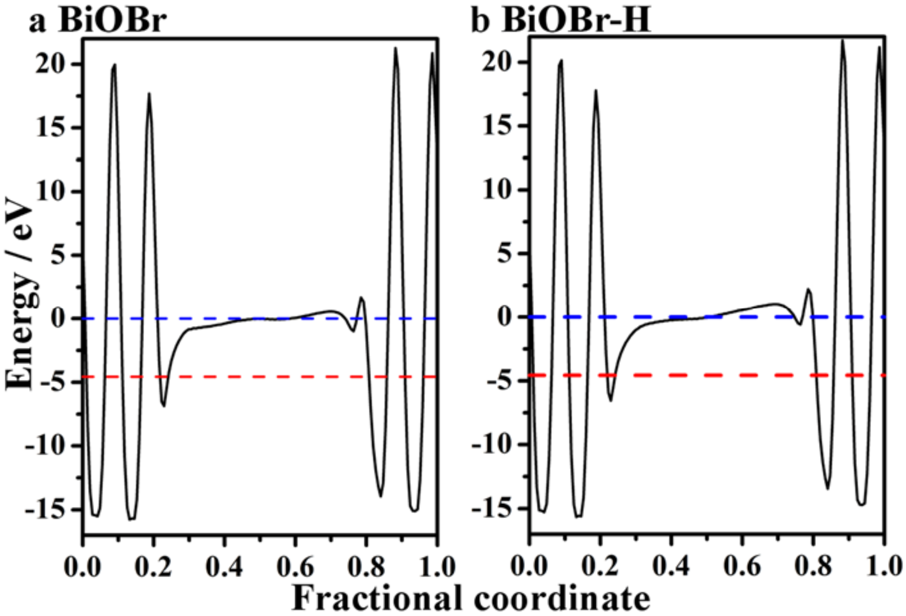

Figure S18. The work functions for BiOBr (a) and BiOBr-H (b), the dashed blue line represents the vacuum level and the dashed red line denotes the Fermi level.

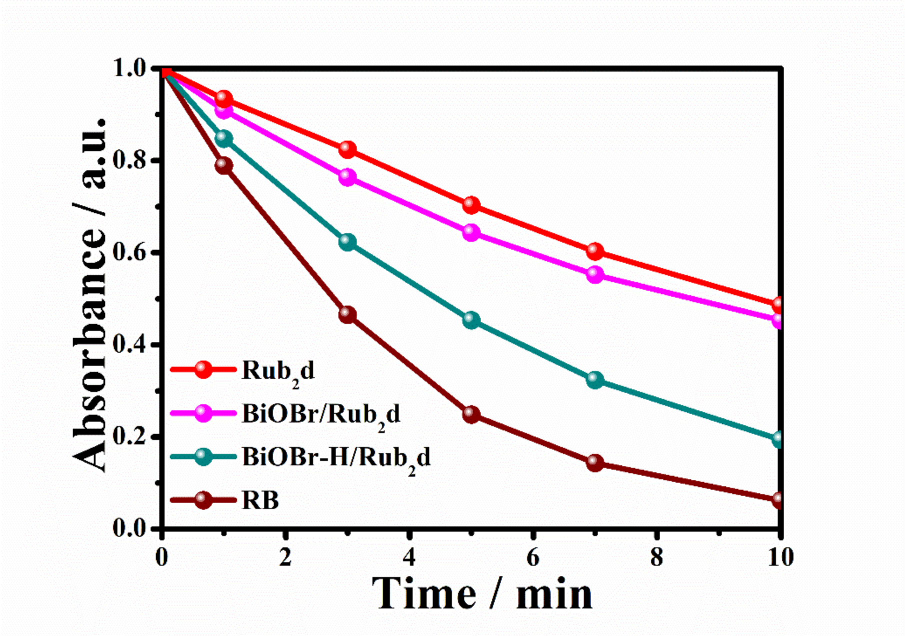

Figure S19. Plots showing the degradation DPBF at 410 nm in the presence of Rub2d, BiOBr/Rub2d, BiOBr-H/Rub2d and Rose Bengal (RB) under light irradiation (520±15 nm).

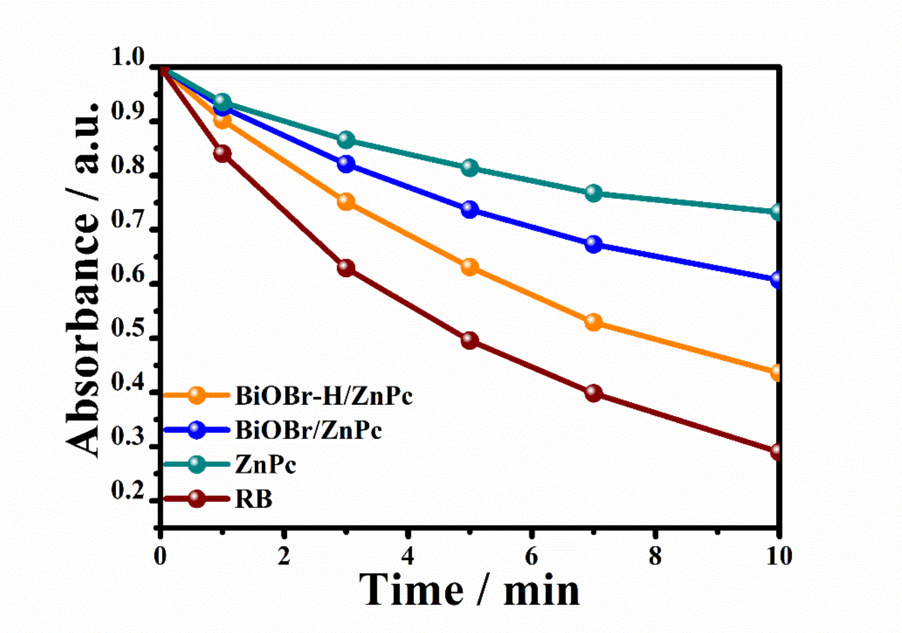

Figure S20. Plots showing the degradation of DPBF at 410 nm in the presence of ZnPc, BiOBr/ZnPc, BiOBr-H/ZnPc and Rose Bengal (RB) under light irradiation (600±15 nm).

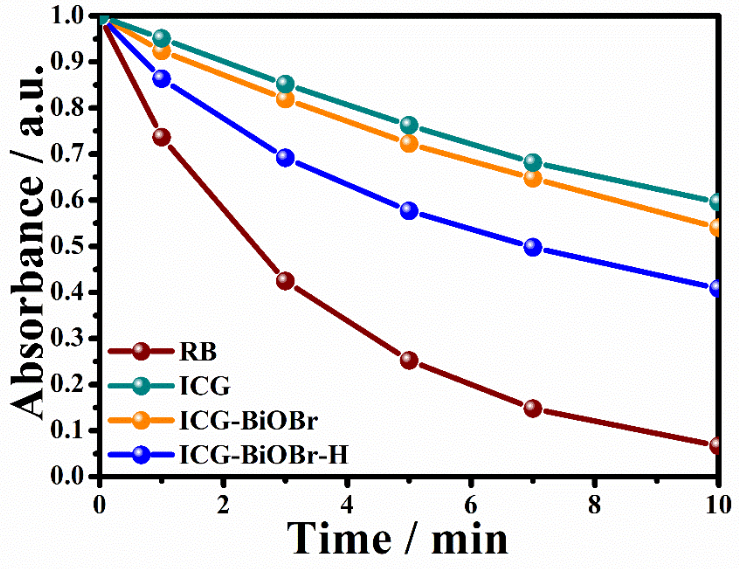

Figure S21. Plots showing the degradation of DPBF at 410 nm in the presence of ICG, BiOBr/ICG, BiOBr-H/ICG and Rose Bengal (RB) under light irradiation (800±15 nm).

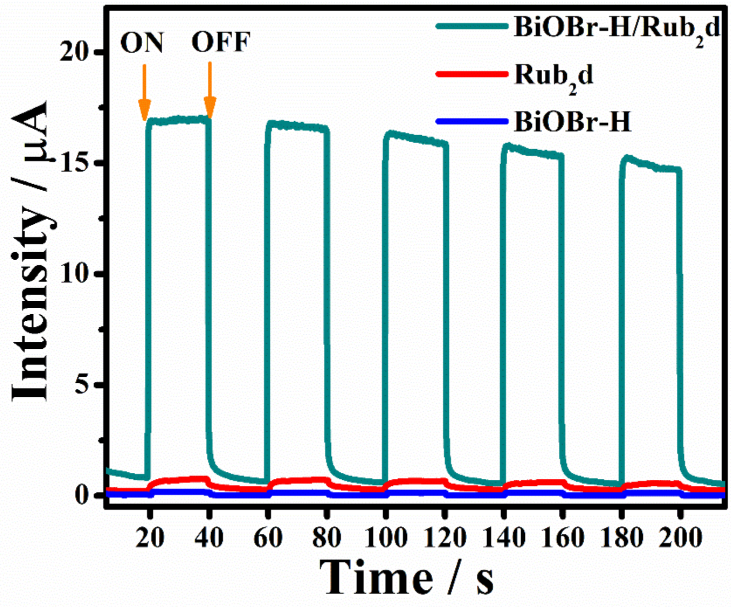

Figure S22. The photocurrent response of BiOBr-H, Rub_2d and BiOBr-H/Rub_2d under Xe lamp excitation (520 nm).

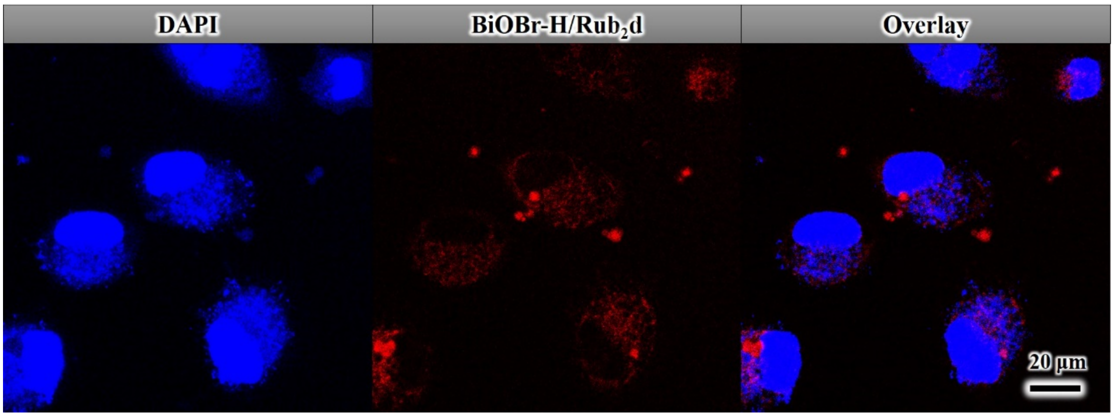

Figure S23. Confocal imaging analysis of HepG-2 cells incubated with BiOBr-H/Rub$_2$d.

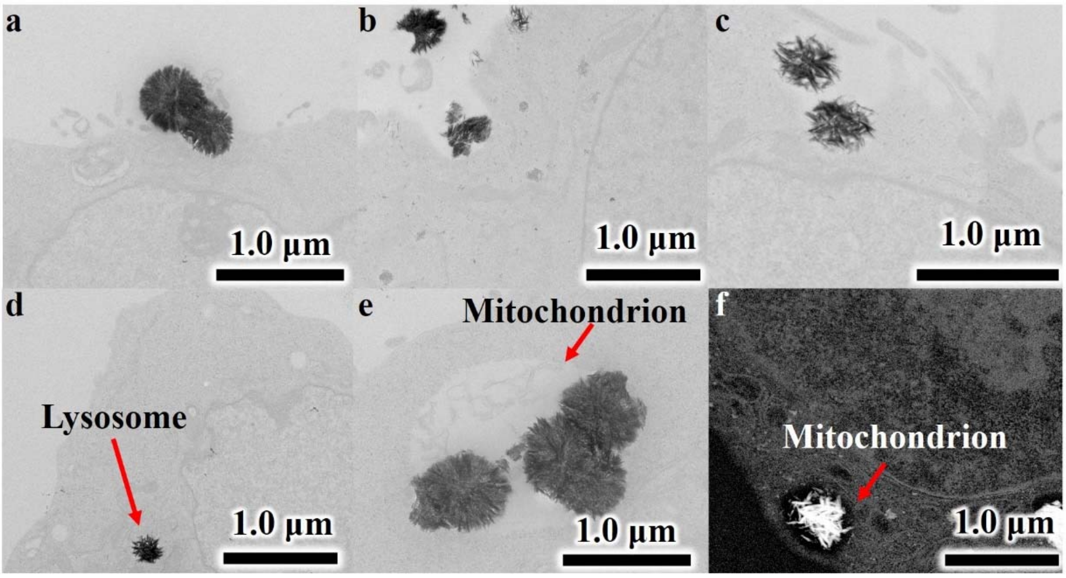

Figure S24. (a-e) TEM and (f) HRTEM of BiOBr-H/Rub$_2$d inside the Hela cells.

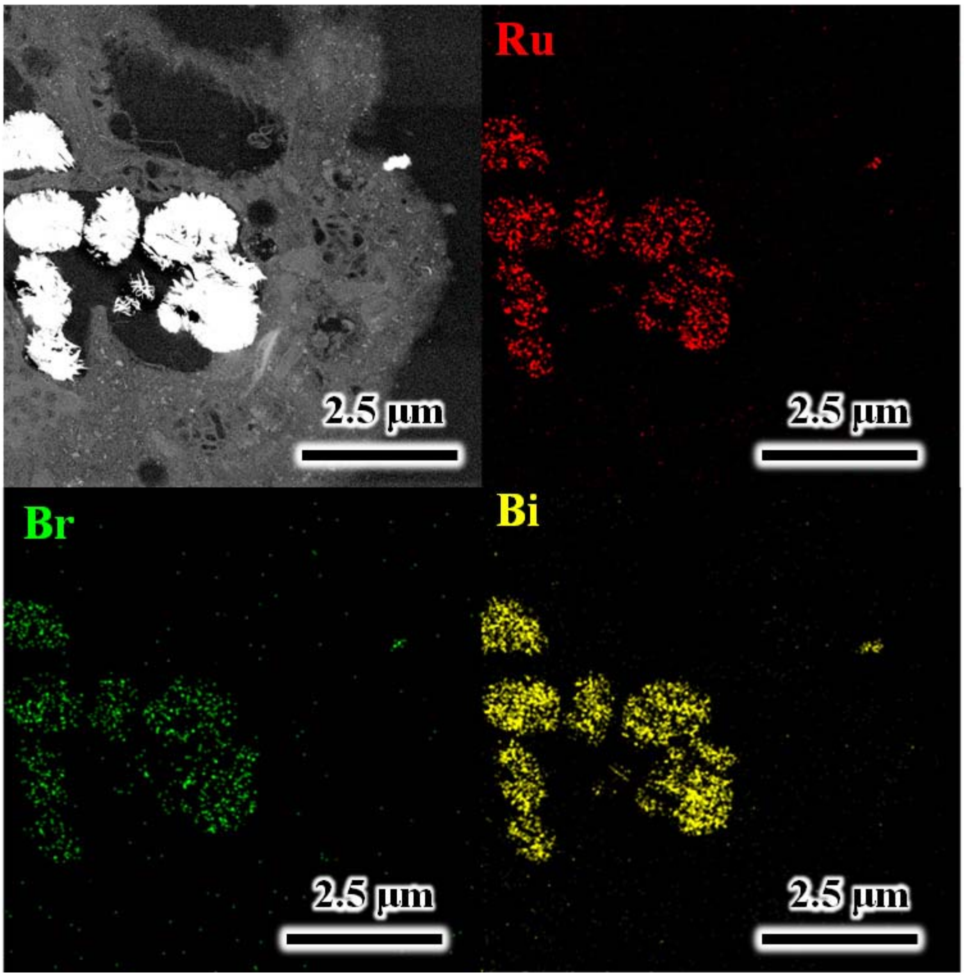

Figure S25. HRTEM and element maps for BiOBr-H/Rub$_2$d inside the Hela cells (incubated with BiOBr-H/Rub$_2$d for 24 h).

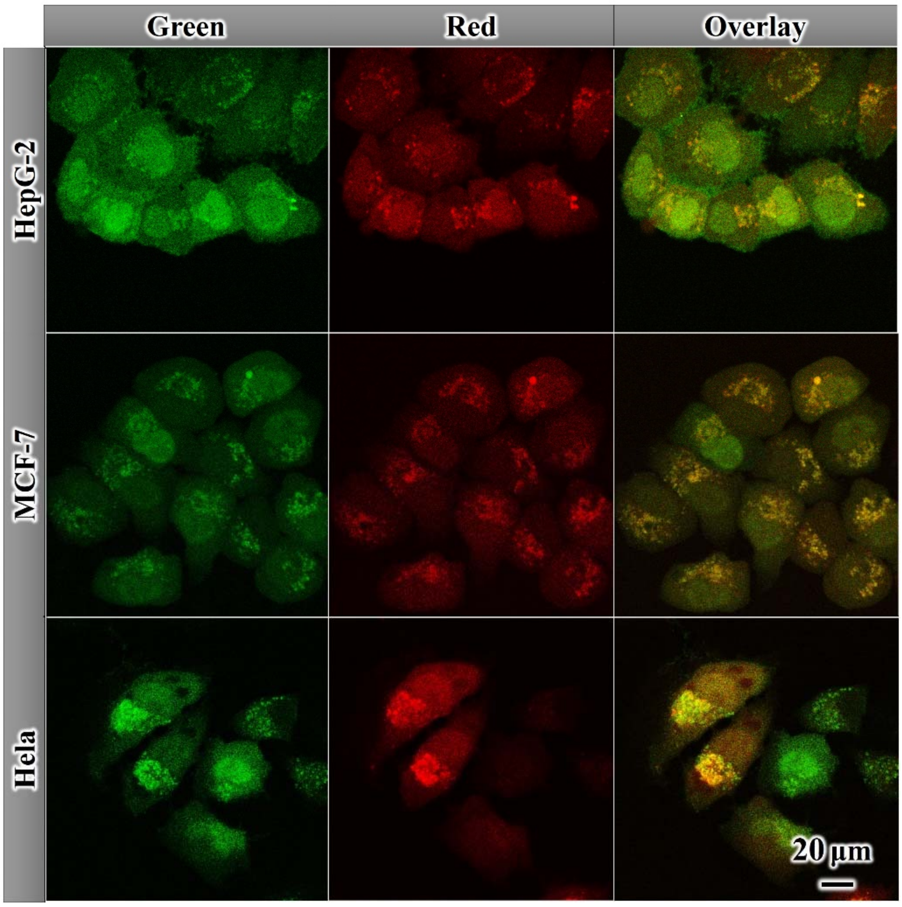

Figure S26. Two-photon fluorescence images of HepG-2, MCF-7 and Hela cells incubated with BiOBr-H/Rub2d under 800 nm excitation.

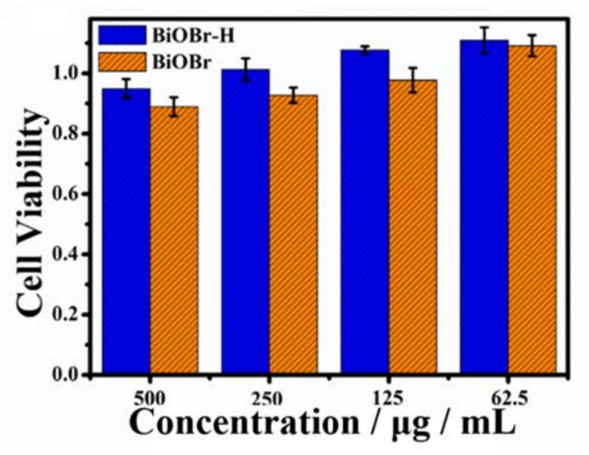

Figure S27. Cell viability of Hela cells incubated with BiOBr, BiOBr-H for 24 h.

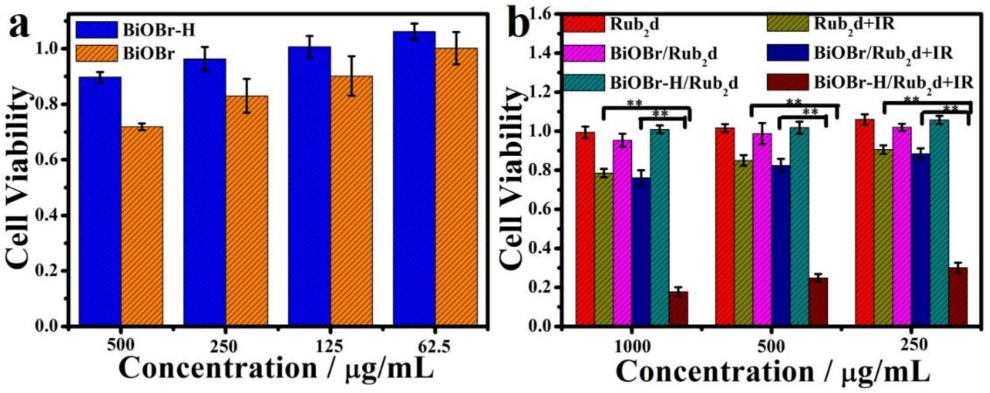

Figure S28. (a) Cell viability of MCF-7 cells incubated with BiOBr, BiOBr-H for 24 h. (b) Cell viability of MCF-7 cells incubated Rub2d, BiOBr-H/Rub2d or BiOBr/Rub2d for 24 h with and without irradiation, respectively. The viability was the average of six measurements (n = 6) and the error bars indicate the standard deviation. *$p < 0.05$, **$p < 0.01$.

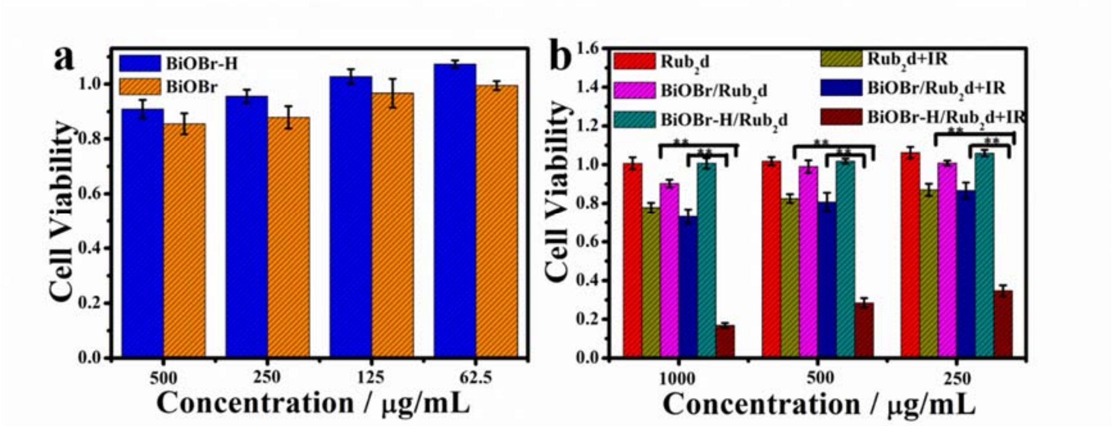

Figure S29. (a) Cell viability of HepG-2 cells incubated with BiOBr, BiOBr-H for 24 h. (b) Cell viability of HepG-2 cells incubated Rub2d, BiOBr-H/Rub2d or BiOBr/Rub2d for 24 h with and without irradiation, respectively. The viability was the averaged of six measurements (n = 6) and the error bars indicate the standard deviation. *$p < 0.05$, **$p < 0.01$.

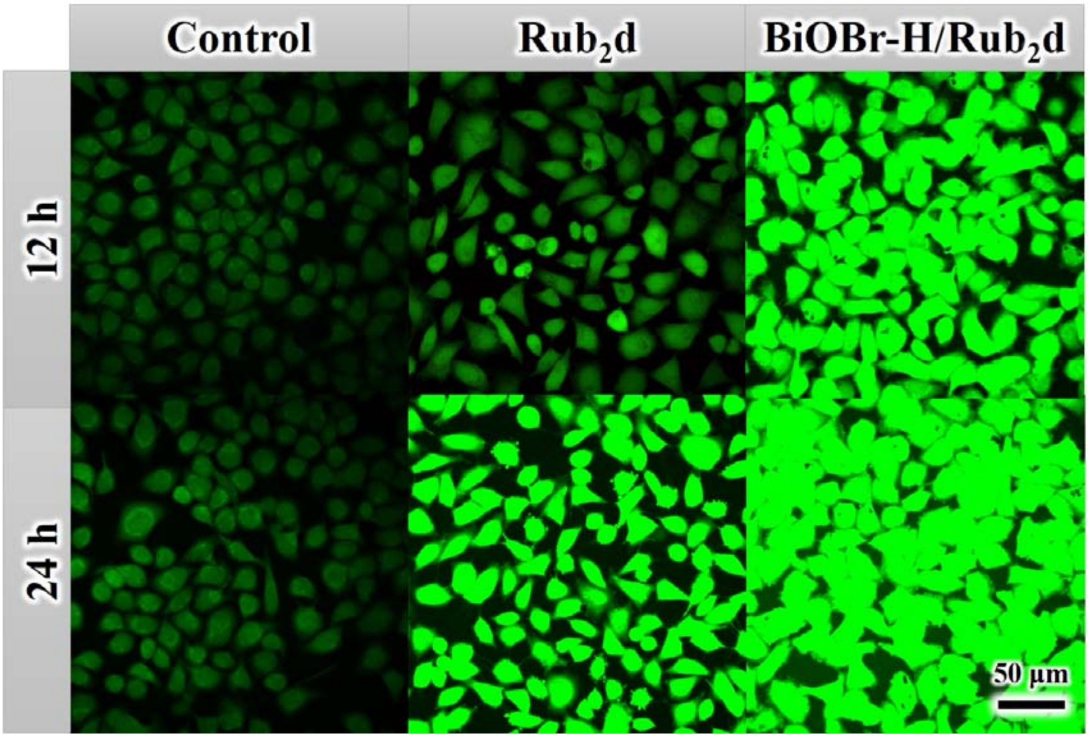

Figure S30. ROS images inside Hela cells after the treatment with Rub$_2$d and BiOBr-H/Rub$_2$d for different time with incubation.

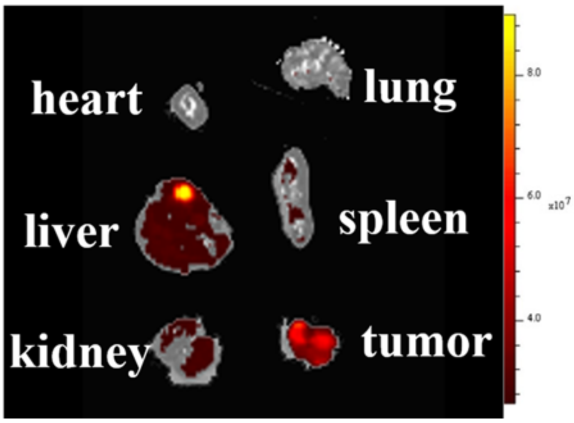

Figure S31. *Ex vivo* fluorescence images of major organs and the tumor after the i.v injection of BiOBr-H/Rub$_2$d for 10 min.

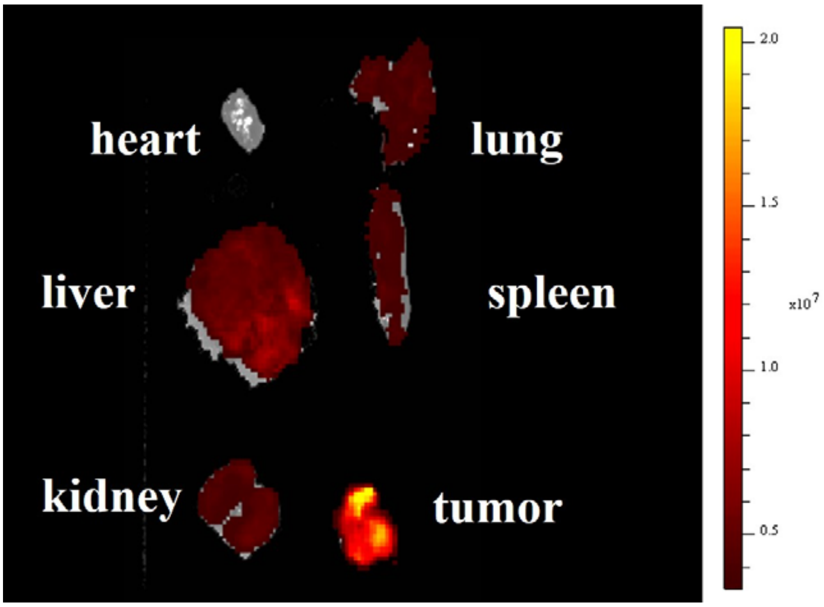

Figure S32. *Ex vivo* fluorescence images of major organs and the tumor after the i.v injection of BiOBr-H/Rub_2d for 24h.

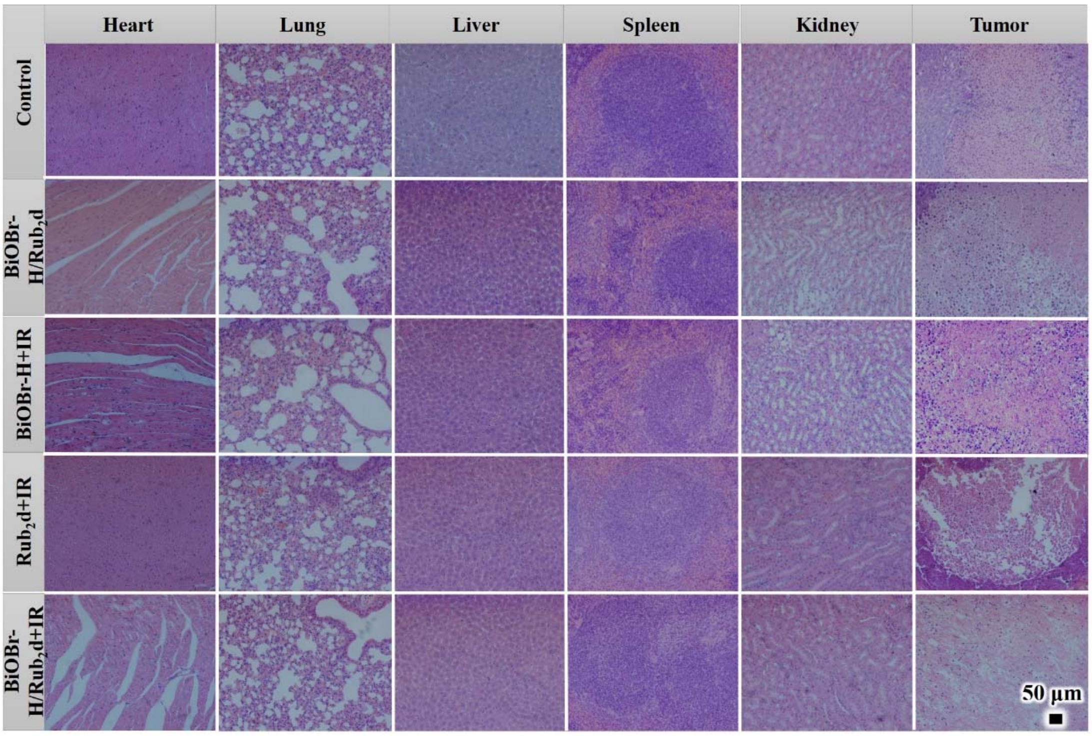

Figure S33. Histology staining of heart, liver, spleen, lung, kidney and tumor slices collected from different groups of mice after 20 days' treatment.

Supplementary Tables

Table S1. Elemental compositions of various samples by XPS.

Samples	C	N	O	Cl	Br	Ru	Bi
Rub$_2$d	70.4 %	9.9 %	13.0 %	2.7 %	–	4.0 %	–
BiOBr	55.6%	5.1%	22.7%	–	8.4%	–	8.2%
BiOBr-H	23.6%	0%	28.6%	–	27.4%	–	20.4%
BiOBr-H/Rub$_2$d	45.7%	3.9%	23.8%	–	13.0%	0.9%	12.7%

Table S2. Fluorescence decay data for Rub$_2$d and BiOBr-H/Rub$_2$d.

Samples	τ_i(ns)[a]	A_i (%)	$\langle\tau\rangle$(ns)	χ^{2}[b]
Rub$_2$d	150.533	24.368	234.47	1.356
	261.520	75.632		
BiOBr-H/Rub$_2$d	164.563	9.253	309.44	1.128
	324.214	90.747		

[a] τ_i (i =1, 2) is the fitted fluorescence lifetime. A_i is the percentage of τ_i. In this case, $\langle\tau\rangle = \Sigma A_i\tau_i$; ΣA_i =1). [b] The goodness of fit is indicated by the value of χ^2.

Table S3. The energy and the surface energy (γ) of the low-index facets of BiOBr.

facet	energy / eV	γ / J·m^{-2}
(001)	−15250.0503	1.5671
(010)	−15248.5691	0.8507
(100)	−15237.7627	1.5306
(011)	−15254.5333	0.4280
(101)	−15236.4387	1.4526
(110)	−15241.6625	0.9086
(111)	−15241.4773	0.8252

Table S4. The quantum yield of singlet oxygen for various samples.

Samples	The quantum yield
Rub_2d	0.22
$BiOBr/Rub_2d$	0.24
$BiOBr\text{-}H/Rub_2d$	0.49
ZnPc	0.31
BiOBr/ZnPc	0.35
BiOBr-H/ZnPc	0.50
ICG	0.15
BiOBr/ICG	0.19
BiOBr-H/ICG	0.28
$Ru(C\text{-}bpy)_2/mLDH$	0.28[10]

References

1. F. A. Bannister, *Mineral. Mag.*, 1935, **24**, 49–58.
2. B. Delley, *J. Chem. Phys.*, 2000, **113**, 7756–7764.
3. J. P. Perdew, K. Burke and M. Ernzerhof, *Phys. Rev. Lett.*, 1996, **77**, 3865–3868.
4. J. Gao, Y. Zheng, Y. Tang, J.-M. Jehng, R. Grybos, J. Handzlik, I. E. Wachs and S. G. Podkolzin, *ACS Catal.*, 2015, **5**, 3078–3092.
5. D. Santos-Carballal, A. Roldan, R. Grau-Crespo and N. H. de Leeuw, *Phys. Chem. Chem. Phys.*, 2014, **16**, 21082–21097.
6. S.-M. Xu, T. Pan, Y.-B. Dou, H. Yan, S.-T. Zhang, F.-Y. Ning, W.-Y. Shi and M. Wei, *J. Phys. Chem. C*, 2015, **119**, 18823–18834.
7. D. K. Kanan and E. A. Carter, *J. Phys. Chem. C*, 2012, **116**, 9876–9887.
8. G. Hu, Q. Tang, D. Lee, Z. Wu and D.-e. Jiang, *Chem. Mater.*, 2017, **29**, 4840–4847.
9. F. Gao, M. Sun, W. Ma, X. Wu, L. Liu, H. Kuang and C. Xu, *Adv. Mater.*, 2017, **29**, 1606864.
10. S. Guan, D. Yang, Y. Weng, H. Lu, X. Meng, X. Qu and S. Zhou, *Adv. Healthc. Mater.*, 2018, 1701123.
